# Post-Recurrence Outcomes Associated with a Bevacizumab-Containing First-Recurrence Strategy in Glioblastoma: A Propensity-Weighted Single-Center Cohort Study with Competing-Risk Analysis

**DOI:** 10.3390/cancers18162665

**Published:** 2026-08-18

**Authors:** Enes Yeşilbaş, Sema Sezgin Göksu

**Affiliations:** 1 Department of Medical Oncology, Ankara Etlik City Hospital, Ankara 06010, Türkiye; 2 Department of Medical Oncology, Faculty of Medicine, Akdeniz University, Antalya 07070, Türkiye; semasezgingoksu@gmail.com

**Keywords:** glioblastoma, bevacizumab, neoplasm recurrence, local, propensity score, competing risks, progression-free survival, neutrophils

## Abstract

**Background/Objectives:** Bevacizumab is widely used at recurrence in glioblastoma, but comparisons are complicated by multimodal treatment selection and progression assessment. We examined outcomes associated with a bevacizumab-containing first-recurrence strategy and explored the components of post-recurrence progression-free survival (prPFS). **Methods:** This retrospective single-center cohort included 166 patients at first recurrence: 114 received a bevacizumab-containing strategy and 52 a non-bevacizumab strategy. Of these, 163 had evaluable outcomes. Local therapy at first recurrence was recorded in 19.3% and 90.4% of the groups, respectively. Treatment strategy was fixed at first recurrence to reduce immortal-time bias. Analyses used pretreatment covariates, multiple imputation, propensity-score overlap weighting, robust variance estimation, and competing-risk methods. Complete-case adjusted models included 117 patients. Recurrence-specific performance status, corticosteroid exposure, and MGMT status were unavailable. **Results:** Among 163 patients, 136 deaths and 145 prPFS events occurred. In the overlap-weighted analysis after multiple imputation, a bevacizumab-containing strategy was not associated with improved post-recurrence overall survival (OS; HR 1.40, 95% CI 0.93 to 2.10) or prPFS (HR 0.81, 95% CI 0.55 to 1.18). Competing-risk analysis showed a lower cause-specific hazard of documented second progression (HR 0.29, 95% CI 0.16 to 0.53) and a higher cause-specific hazard of death before documented progression, although its CI included the null (HR 1.65, 95% CI 0.93 to 2.93). Higher baseline neutrophil-to-lymphocyte ratio was associated with poorer OS (HR per doubling 1.49, 95% CI 1.20 to 1.85), but treatment interactions were inconsistent across parameterizations. **Conclusions:** A bevacizumab-containing first-recurrence strategy was not associated with improved post-recurrence OS or prPFS. Divergent progression and death patterns caution against interpreting progression-based endpoints as direct evidence of disease control in this observational setting.

## Simple Summary

Bevacizumab is often used when glioblastoma returns after initial treatment. Randomized trials have not shown that it helps patients live longer, and studies based on routine care are difficult to interpret because patients often receive several treatments together. We studied 166 patients whose tumor returned after first-line treatment. We compared patients whose treatment at first recurrence included bevacizumab with those treated in other ways, often including repeat surgery or radiotherapy. Survival after recurrence was not better with the bevacizumab-containing approach. Further tumor growth was recorded less often in these patients, but they more often died before further growth was documented. Because bevacizumab can change how tumors appear on scans, and because death can occur before further growth is recorded, a lower frequency of recorded progression does not necessarily mean better disease control. These findings describe treatment approaches used in routine practice rather than the effect of bevacizumab alone.

## 1. Introduction

Glioblastoma is the most common malignant primary brain tumor in adults, and its prognosis remains poor despite maximal safe resection and chemoradiotherapy [1,2]. Almost all patients relapse, and survival after recurrence is usually measured in months. No single standard treatment exists at recurrence. Guidelines recommend individualized selection among repeat resection, re-irradiation, systemic therapy, and supportive care [3]. Performance status, prior treatment, the interval since radiotherapy, and the feasibility of further local therapy influence both treatment selection and prognosis, making confounding by indication a central challenge in observational studies of recurrent glioblastoma.

The 2021 World Health Organization (WHO) classification distinguishes glioblastoma, IDH-wildtype, from IDH-mutant astrocytomas, reflecting their substantially different natural history [4,5]. Many real-world studies of bevacizumab predated routine molecular classification and either included mixed high-grade glioma populations or reported molecular status incompletely [6]. Molecular markers also remain important beyond diagnostic classification. MGMT promoter methylation predicts benefit from temozolomide and is also associated with a more favorable prognosis, with a reported hazard ratio of 0.45 (95% CI, 0.32 to 0.61) independent of treatment [7]. Incomplete molecular characterization can therefore leave prognostic heterogeneity even within a single diagnostic category.

Bevacizumab targets vascular endothelial growth factor. An early phase 2 study of bevacizumab with irinotecan reported substantial radiographic responses in recurrent glioblastoma [8]. Randomized trials have not shown an overall survival benefit, either in newly diagnosed disease [9,10] or at recurrence, where EORTC 26101 found no overall survival advantage although the addition of bevacizumab did not adversely affect health-related quality of life [11,12]. Pseudoresponse remains an important challenge when interpreting progression-based outcomes. Antiangiogenic therapy reduces vascular permeability and contrast enhancement without a proportional reduction in viable tumor, potentially obscuring or delaying radiographic evidence of progression [13]. Response Assessment in Neuro-Oncology criteria address this in part by incorporating non-enhancing disease, but assessment during antiangiogenic therapy remains difficult [14].

A further distinction is needed between drug efficacy and treatment-strategy effectiveness. Trials compare defined regimens under protocol conditions, whereas treatment at recurrence in routine care is often multimodal, with systemic therapy, repeat surgery, and re-irradiation selected together. Observational comparisons therefore estimate associations between treatment strategies as delivered rather than the isolated pharmacologic effect of a single drug. This distinction is particularly relevant when the compared strategies differ substantially in their use of local therapy. Earlier retrospective studies have also differed in diagnostic era and in the recurrence point used to define exposure [15,16], creating additional potential for treatment-selection and immortal-time biases [17].

The study population comprised patients assigned to the WHO 2021 integrated diagnostic category of glioblastoma, IDH-wildtype, with treatment-strategy classification anchored to the first documented recurrence. Adjustment was limited to characteristics available before treatment-strategy assignment, and propensity-score overlap weighting with multiple imputation was used to address measured treatment-selection imbalance and missing covariate data. The objective was to examine the association of a bevacizumab-containing first-recurrence strategy with post-recurrence overall survival and post-recurrence progression-free survival (prPFS). Because prPFS combines documented second progression and death, these components were analyzed separately in a supportive competing-risk analysis. Baseline neutrophil-to-lymphocyte ratio (NLR) was assessed as a prognostic factor, with its interaction with treatment strategy considered exploratory.

## 2. Materials and Methods

### 2.1. Study Design and Patient Population

**Figure 1:**
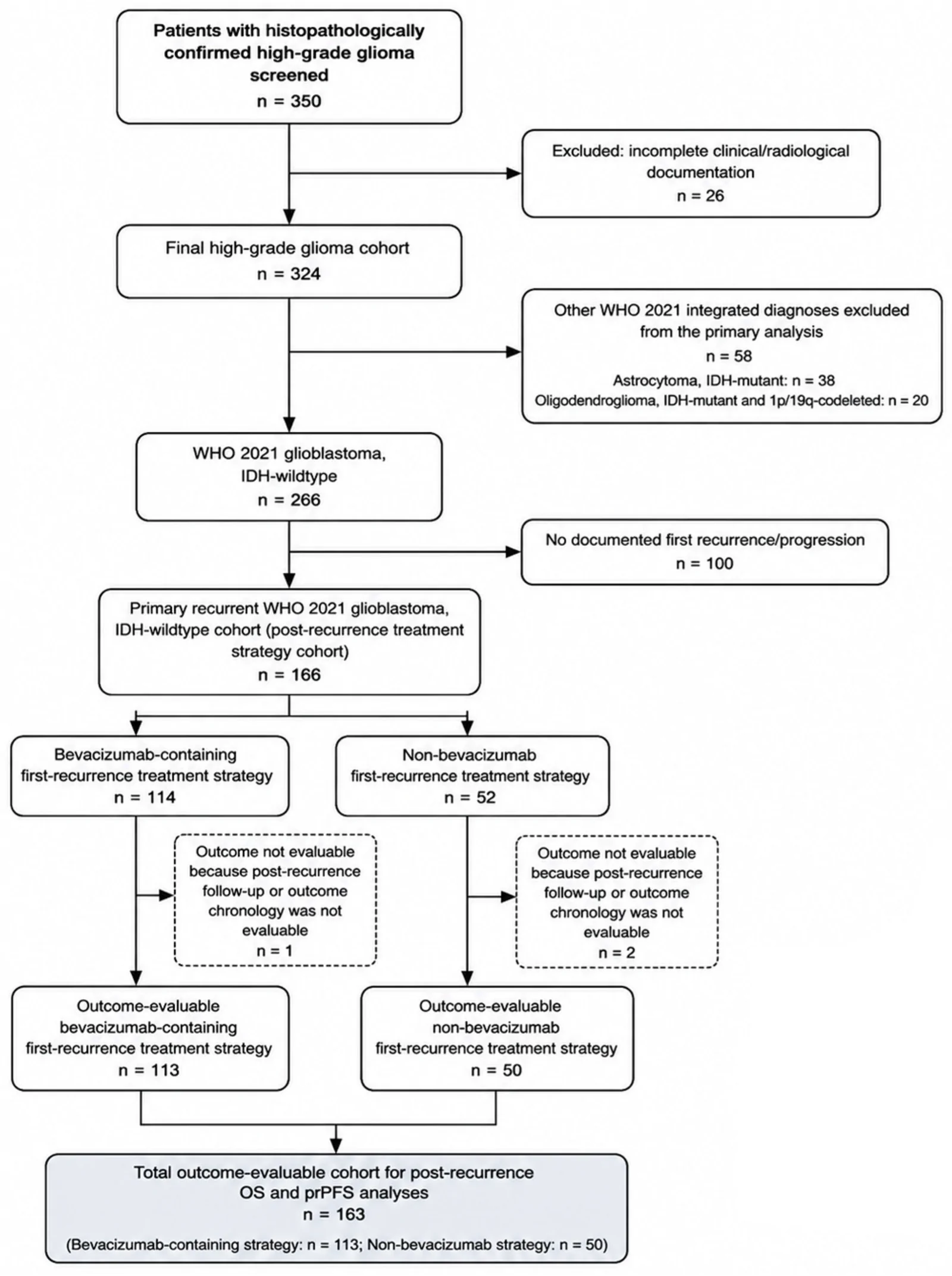
Study flow diagram for derivation of the analysis cohort. Patients with histopathologically confirmed high-grade glioma were screened and mapped to WHO 2021 integrated diagnostic categories using the molecular information available in the clinical record. The primary recurrent cohort was restricted to patients assigned to glioblastoma, IDH-wildtype with a documented first recurrence or progression. A bevacizumab-containing first-recurrence strategy was defined by the presence of bevacizumab in the recorded first-recurrence treatment strategy. Abbreviations: IDH, isocitrate dehydrogenase; OS, overall survival; prPFS, post-recurrence progression-free survival; WHO, World Health Organization.

This was a retrospective, single-center cohort study conducted at the Department of Medical Oncology, Akdeniz University Faculty of Medicine, and reported in accordance with the STROBE statement for cohort studies [18]. A completed STROBE checklist is provided as a separate Supplementary File. Consecutive patients with histopathologically confirmed high-grade glioma diagnosed between January 2010 and December 2022 were screened, and follow-up data were updated through 15 June 2023. Data were extracted from electronic medical records, including pathology reports, radiology reports, and hospital mortality and follow-up records. The same data sources and extraction procedures were used for both treatment-strategy groups, although completeness varied for some variables. The derivation of the analysis cohort is summarized in Supplementary Table S1 and shown in Figure 1.

Available pathological and molecular data were retrospectively mapped to WHO 2021 diagnostic categories [4]. Classification was based on the final integrated neuropathological report together with molecular results recorded in the clinical record, primarily IDH mutation and 1p/19q codeletion status. Molecular testing was neither uniform nor complete across the study period. Diagnostic assignment therefore reflects the integrated neuropathological report and the molecular information available in the clinical record rather than complete contemporary histomolecular confirmation.

The structured dataset did not capture the method of IDH assessment or all histological and molecular features required to reconstruct contemporary WHO 2021 confirmation, including histological grade 4 features, EGFR amplification, and combined chromosome 7 gain with chromosome 10 loss; TERT promoter status was also unavailable. Such a subgroup could therefore not be reliably reconstructed. Among the 58 tumors assigned to an IDH-mutant integrated diagnostic category, 45 had a directly recorded IDH1-mutant result and 13 had no structured IDH1 result. Integrated diagnostic categories should accordingly not be read as counts of directly documented test results. Among the 266 tumors assigned to glioblastoma, IDH-wildtype, 123 (46.2%) had a directly recorded IDH1 result of wild-type or not mutant, and the remaining 143 had no structured IDH1 result. MGMT promoter methylation was not available in the structured dataset and could not be included in prognostic adjustment. Diagnostic reclassification, marker availability, and the reconciliation of structured IDH1 results with integrated diagnoses are detailed in Supplementary Tables S2 and S3.

Of 350 patients screened, 26 were excluded because of incomplete clinical or radiological documentation, leaving 324 patients. Of these, 266 were assigned to the integrated diagnostic category of glioblastoma, IDH-wildtype; the remaining 58 were classified as astrocytoma, IDH-mutant (*n* = 38) or oligodendroglioma, IDH-mutant and 1p/19q-codeleted (*n* = 20) and were excluded. Among the 266 patients assigned to glioblastoma, IDH-wildtype, 100 had no documented first recurrence or progression and therefore could not contribute to a first-recurrence comparison (Supplementary Table S4). The primary recurrent cohort therefore included 166 patients: 114 (68.7%) with a bevacizumab-containing first-recurrence strategy and 52 (31.3%) with a non-bevacizumab strategy. Three patients lacked evaluable post-recurrence outcomes, leaving 163 patients for survival analyses.

Fourteen tumors previously diagnosed as grade 3 anaplastic astrocytoma were assigned to the integrated category of glioblastoma, IDH-wildtype and WHO grade 4. All 14 had an IDH1 result recorded as wild-type or not mutant in the structured dataset, although the available data were insufficient to reconstruct the complete histomolecular basis for this reclassification. Twelve of these patients entered the primary recurrent cohort and were retained in the main analysis; their exclusion was examined in a sensitivity analysis.

### 2.2. Treatment Strategy Definition

Patients were classified as receiving a bevacizumab-containing first-recurrence strategy if bevacizumab was recorded as part of the treatment strategy at the first documented recurrence; all other first-recurrence strategies were classified as non-bevacizumab. Treatment-strategy classification was fixed at the first documented recurrence and was not changed by treatments administered later in the recurrence course. Anchoring classification to the first documented recurrence was intended to approximate the treatment decision at recurrence and to reduce the immortal-time bias associated with defining exposure by treatment received at any later point in the disease course [17].

Treatment components recorded as part of the first-recurrence strategy were not required to have been initiated simultaneously. If more than one modality was recorded as part of the first-recurrence strategy, all recorded components were retained in the description of that strategy, while the presence of bevacizumab determined group assignment regardless of treatment sequence. For patients assigned to a bevacizumab-containing strategy, the interval from first documented recurrence to bevacizumab initiation was calculated when both dates were available and chronologically valid. A comparable recurrence-to-treatment interval could not be derived for the non-bevacizumab strategy because a uniform treatment-initiation date was not available across its heterogeneous modalities, including surgery, re-irradiation, and systemic therapy; treatment-initiation dates were not imputed.

### 2.3. Endpoints and Radiological Assessment

The primary endpoint was post-recurrence overall survival (OS), measured from the first documented recurrence or progression (hereafter, first recurrence) to death from any cause, with censoring at the date of last follow-up. The secondary endpoint was post-recurrence progression-free survival (prPFS), measured from first recurrence to the earlier of documented second progression or death from any cause. Patients with neither event were censored at last follow-up. Because prPFS is a composite endpoint, each patient was additionally classified as having documented second progression, death before documented second progression, or censoring without either event. Three recorded second-progression dates preceded the corresponding first-recurrence date and were considered chronologically invalid. These progression dates were disregarded, and all three patients remained in the prPFS analysis with their subsequent valid death dates treated as prPFS events. They were classified in the death before documented second progression category. The term prPFS is used for this study-specific post-recurrence endpoint and should not be confused with the conventional trial endpoint PFS2, which is measured from randomization to progression on a subsequent treatment line or death.

Progression dates were derived from contemporaneous clinical and radiological records and reflected the assessment made during routine care, using criteria consistent with the Response Assessment in Neuro-Oncology framework [14]. No central or blinded radiological re-review was performed, and surveillance imaging followed routine practice rather than protocol-defined intervals; exact imaging intervals were not available as a structured variable for analysis. Corticosteroid use and dose at the time of recurrence or imaging could not be reconstructed reliably, and non-enhancing progression was not separately coded. Retrospective adjudication of possible pseudoprogression was therefore not possible, and some misclassification of progression cannot be excluded.

Because antiangiogenic therapy can alter contrast enhancement [13], documented progression may have been ascertained differently between treatment strategies in ways that could not be quantified from the available data. A supportive competing-risk analysis was therefore used to characterize documented second progression and death before documented second progression separately, in addition to the composite prPFS endpoint.

### 2.4. Statistical Analysis

Continuous variables were summarized as medians with interquartile ranges (IQRs) and categorical variables as counts and percentages. Continuous variables were compared using the Mann–Whitney U test and categorical variables using the chi-square or Fisher exact test. Standardized mean differences (SMDs) were used to quantify covariate imbalance, with an absolute value of 0.10 or greater considered indicative of potentially meaningful imbalance [19]; for multilevel variables, the largest absolute SMD across categories is reported. Survival was estimated using the Kaplan–Meier method.

The comparative analyses addressed the association between initiation of a bevacizumab-containing and a non-bevacizumab first-recurrence strategy in the population with the greatest overlap in measured pretreatment characteristics [20]. They were not designed to isolate the pharmacologic effect of bevacizumab from irinotecan or from concurrent local therapy. Pretreatment-adjusted Cox models included treatment strategy, age at first recurrence, ECOG performance status at diagnosis, baseline NLR on the log2 scale, time from diagnosis to first recurrence, tumor size, multicentric disease, and initial extent of resection. ECOG performance status at diagnosis was used because performance status at recurrence was not uniformly documented. Repeat surgery and re-irradiation were treated as components of the recurrence-directed strategy and were not used as adjustment covariates. The proportional hazards assumption was examined using Schoenfeld residuals. When covariate-specific evidence of non-proportionality was identified, sensitivity models allowed the affected covariates to vary with log-transformed follow-up time, while the treatment-strategy coefficient was retained as the parameter of interest. Tied event times were handled with the Breslow method.

Missing covariate values were addressed by multiple imputation by chained equations using fully conditional specification among the 163 patients with evaluable outcomes, generating 50 completed datasets. Continuous variables were imputed using predictive mean matching and binary variables using logistic regression. Treatment strategy, observed pretreatment covariates, outcome indicators, and Nelson–Aalen cumulative-hazard estimates were included as predictors in the imputation models [21]; survival times and event indicators were not imputed. Age at first recurrence was recalculated from age at diagnosis and the observed or imputed diagnosis-to-recurrence interval rather than imputed directly. Missing-data patterns and handling are summarized in Supplementary Table S5.

Propensity scores for a bevacizumab-containing strategy were estimated by logistic regression. The propensity-score model included age at first recurrence, sex, ECOG performance status at diagnosis, baseline log2-transformed NLR, time from diagnosis to first recurrence, initial non-gross total resection, tumor size, multicentric disease, tumor location, and seizure at presentation. Propensity scores and overlap weights were re-estimated independently within each completed dataset, and the resulting treatment-effect estimates were combined using Rubin’s rules [22]. The functional form of continuous covariates was examined using restricted cubic splines. Overlap weights were calculated as 1 minus the propensity score for the bevacizumab-containing group and as the propensity score for the non-bevacizumab group [23]. Balance was assessed using absolute SMDs, and positivity was assessed using the propensity-score and weight distributions, the empirical common-support interval, and the effective sample size after weighting [24]. Weighted Cox models used robust sandwich variance estimation. Complete-case analyses were retained as sensitivity analyses and are reported alongside the multiply imputed estimates.

Absolute effects were summarized as overlap-weighted survival probabilities at 6, 12, 18, and 24 months, as between-strategy differences in percentage points, and as differences in restricted mean survival time at the same horizons, the last providing a summary that does not depend on proportional hazards. Confidence intervals for these quantities were obtained using 2000 stratified patient-level bootstrap resamples, with resampling performed separately within treatment-strategy groups. The same resampled patient indices were applied across the 50 completed datasets, and the propensity-score model and overlap weights were re-estimated within each completed dataset in every bootstrap sample. Percentile bootstrap confidence intervals are reported.

Documented second progression and death before documented second progression were analyzed as mutually competing events. Overlap-weighted cumulative incidence functions were estimated using the Aalen–Johansen estimator separately within each completed dataset and averaged across the 50 imputations [25]. Between-strategy differences in cumulative incidence at 6, 12, 18, and 24 months were estimated using the same stratified patient-level bootstrap framework described above. Overlap-weighted cause-specific Cox models were used to estimate hazard ratios separately for documented second progression and death before documented second progression.

Baseline NLR was modeled continuously both per raw-unit increase and after log2 transformation, for which hazard ratios represent the association with a doubling of NLR. Potential nonlinearity was assessed using restricted cubic splines with four knots placed at the 5th, 35th, 65th, and 95th percentiles. Overall NLR association and departure from linearity were assessed using likelihood-ratio tests comparing nested Cox models. Effect modification was examined using treatment-strategy-by-NLR interaction terms on both the raw and log2-transformed scales. The historical cutoff of 3.81 was retained only as a sensitivity analysis because the original cutoff-selection procedure could not be independently reverified from the archived statistical documentation.

Sensitivity to unmeasured confounding for the principal overlap-weighted OS and prPFS estimates after multiple imputation was assessed using E-values for the point estimates and for the confidence limits closest to the null, with hazard ratios converted to the risk-ratio scale using the approximation for common outcomes [26]. Sensitivity analyses included complete-case and multiply imputed pretreatment-adjusted Cox models; complete-case and multiply imputed pretreatment-only overlap-weighted models; exclusion of tumors reclassified from a previous grade 3 diagnosis to WHO 2021 grade 4; separate exclusion of patients with recurrence within 6 months and within 3 months of diagnosis; and restriction of the bevacizumab-containing group to bevacizumab plus irinotecan regimens. The early-recurrence exclusion analyses were restricted to patients with an observed and chronologically valid diagnosis-to-recurrence interval and used complete-case pretreatment-adjusted Cox models. All tests were two-sided, with *p* < 0.05 considered statistically significant. No adjustment was made for multiplicity; interaction, competing-risk, RMST, subgroup, and sensitivity analyses were considered exploratory unless otherwise specified. Analyses were performed using IBM SPSS Statistics version 25.0 and Python version 3.12 with pandas, NumPy, SciPy, statsmodels, and scikit-learn.

## 3. Results

### 3.1. Cohort and Baseline Characteristics

Baseline and first-recurrence characteristics of the primary recurrent cohort (*n* = 166) are summarized in Table 1.

Patients receiving a bevacizumab-containing strategy were older at diagnosis (median 55.0 versus 48.0 years; SMD 0.598) and at first recurrence (55.5 versus 51.6 years; SMD 0.473), and had a shorter interval from diagnosis to first recurrence (9.2 versus 12.0 months; SMD 0.566). Neurological sequelae were recorded more often in the non-bevacizumab group (63.5% versus 44.2%; SMD 0.393). Additional imbalances above the 0.10 threshold were observed for initial extent of resection, tumor size, tumor location, seizure at presentation, and baseline NLR (Table 1).

The largest between-group differences involved treatment-related characteristics after recurrence rather than pretreatment characteristics. Repeat surgery was performed in 82.7% of patients receiving a non-bevacizumab strategy and in 16.7% of those receiving a bevacizumab-containing strategy (SMD 1.758), re-irradiation in 28.8% and 4.4%, respectively (SMD 0.696), and systemic antineoplastic therapy during the recurrence course in 78.8% and 100.0%, respectively (SMD 0.733). These post-recurrence treatment variables are reported descriptively and were not used as adjustment covariates.

### 3.2. First-Recurrence Treatment Strategies

Recorded first-recurrence strategies are detailed in Supplementary Table S6. Among patients receiving a bevacizumab-containing strategy, 85.1% received bevacizumab with irinotecan without recorded local therapy, 10.5% received local therapy together with bevacizumab and irinotecan, and 4.4% received bevacizumab monotherapy or an unspecified bevacizumab-containing regimen. Among patients receiving a non-bevacizumab strategy, 40.4% were treated with repeat surgery plus temozolomide with or without re-irradiation, 38.5% with repeat surgery alone, 11.5% with re-irradiation without surgery or systemic therapy, 7.7% with temozolomide rechallenge or extended temozolomide without local therapy, and 1.9% with irinotecan alone. Any local treatment at first recurrence was recorded in 19.3% of the bevacizumab-containing group and 90.4% of the non-bevacizumab group. These distributions indicate that the comparison involved different multimodal first-recurrence strategies rather than a simple presence-versus-absence contrast for bevacizumab.

A bevacizumab initiation date was recorded for 110 of the 114 patients assigned to a bevacizumab-containing strategy. Two date pairs were chronologically inconsistent and were excluded from this calculation, leaving 108 evaluable patients (94.7% of the group). The median interval from first documented recurrence to bevacizumab initiation was 13.5 days (IQR, 6.0 to 31.3; range, 0 to 197), and treatment was started within 30 days in 74.1%, within 60 days in 87.0%, and within 90 days in 93.5%. Thirty of the 52 patients assigned to a non-bevacizumab strategy (57.7%) received bevacizumab later in the recurrence course; consistent with the strategy definition, this did not alter their assignment. Subsequent treatment lines and attrition after second and later progressions are summarized in Supplementary Table S7.

### 3.3. Post-Recurrence Survival Outcomes

Among the 163 patients with evaluable post-recurrence outcomes, 136 deaths and 145 prPFS events were recorded.

Cox regression analyses for post-recurrence OS are shown in Table 2. In the univariable model, a bevacizumab-containing strategy was associated with a higher hazard of death (HR 1.57, 95% CI 1.08 to 2.29, *p* = 0.017). In the complete-case pretreatment-adjusted model, the estimate was similar but the confidence interval included the null (HR 1.52, 95% CI 0.90 to 2.58, *p* = 0.117). Baseline NLR per doubling (HR 1.49, 95% CI 1.20 to 1.85, *p* < 0.001) and initial non-gross total resection (HR 1.95, 95% CI 1.16 to 3.27, *p* = 0.011) were associated with post-recurrence OS in the adjusted model. Corresponding analyses for prPFS are shown in Supplementary Table S8. There was no evidence of non-proportional hazards for the treatment-strategy variable itself for either endpoint (*p* = 0.293 for post-recurrence OS and *p* = 0.565 for prPFS). Global proportional-hazards diagnostics and the corresponding time-varying covariate sensitivity analyses are reported in Supplementary Table S9.

**Figure 2:**
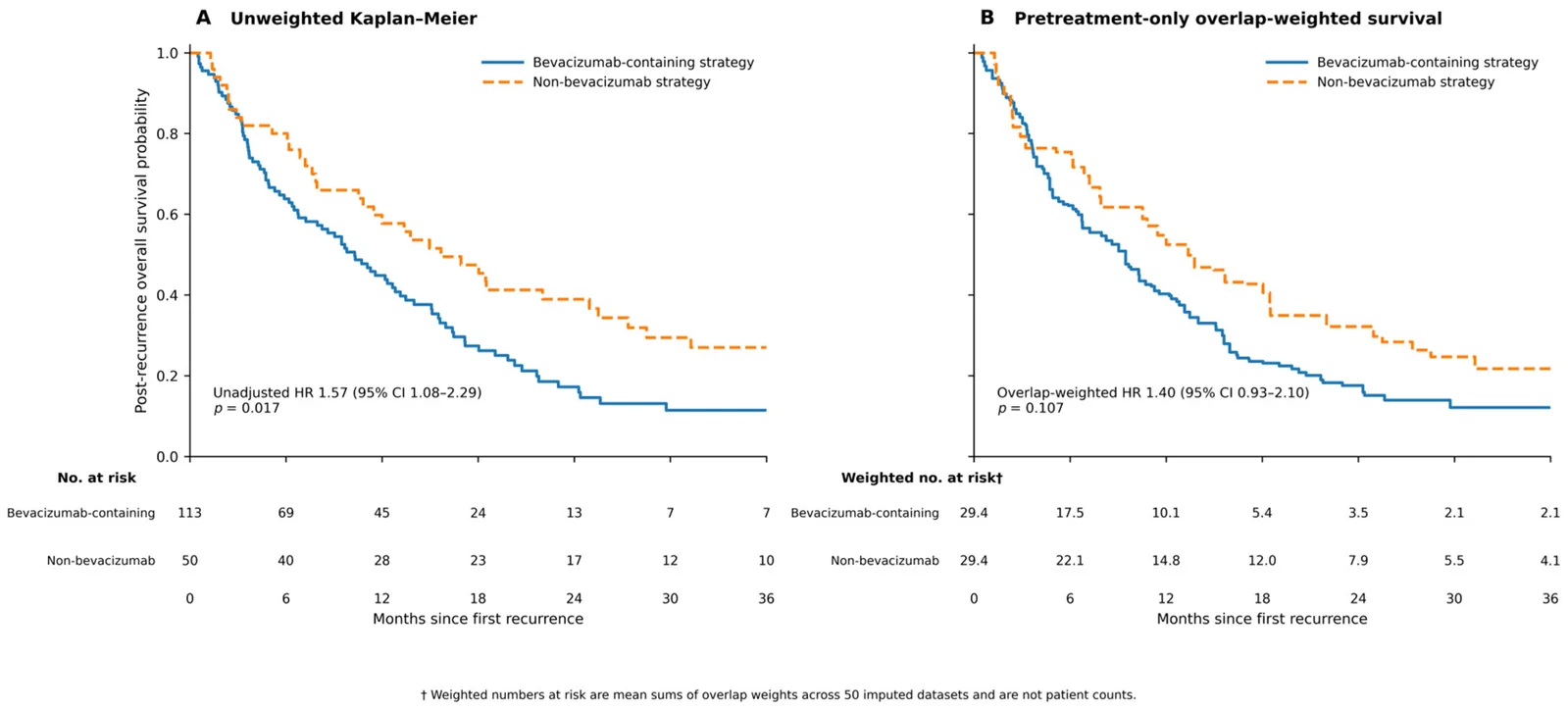
Post-recurrence overall survival according to first-recurrence treatment strategy. Panel (**A**) shows unweighted Kaplan–Meier survival curves in the outcome-evaluable cohort (*n* = 163) with the corresponding unadjusted Cox estimate. Panel (**B**) shows survival curves after pretreatment-only propensity-score overlap weighting, averaged across 50 multiply imputed datasets, with the corresponding pooled overlap-weighted Cox estimate. The propensity-score model included only pretreatment characteristics and was re-estimated separately within each imputed dataset. Numbers at risk in Panel (**A**) represent observed patient counts. Weighted numbers at risk in Panel (**B**) represent the mean sums of overlap weights across the 50 imputed datasets and should not be interpreted as patient counts. The *x*-axis was truncated at 36 months for graphical presentation only; all available follow-up was retained in the Cox analyses. Abbreviations: CI, confidence interval; HR, hazard ratio; OS, overall survival.

Table 3 summarizes the principal comparative estimates. Unweighted and overlap-weighted survival curves for post-recurrence OS are shown in Figure 2A and Figure 2B, respectively. Across the five analyses summarized in Table 3, the hazard ratio for post-recurrence OS was above 1, ranging from 1.34 to 1.57; in the overlap-weighted analysis after multiple imputation it was 1.40 (95% CI, 0.93 to 2.10). Apart from the unadjusted comparison, all confidence intervals in Table 3 included the null. Corresponding unweighted and overlap-weighted prPFS curves are shown in Supplementary Figure S1A,B. For prPFS, the hazard ratio was below 1 across the five analyses, ranging from 0.68 to 0.87, and was 0.81 (95% CI, 0.55 to 1.18) in the overlap-weighted analysis after multiple imputation. All confidence intervals for prPFS in Table 3 included the null.

### 3.4. Covariate Balance and Positivity

**Figure 3:**
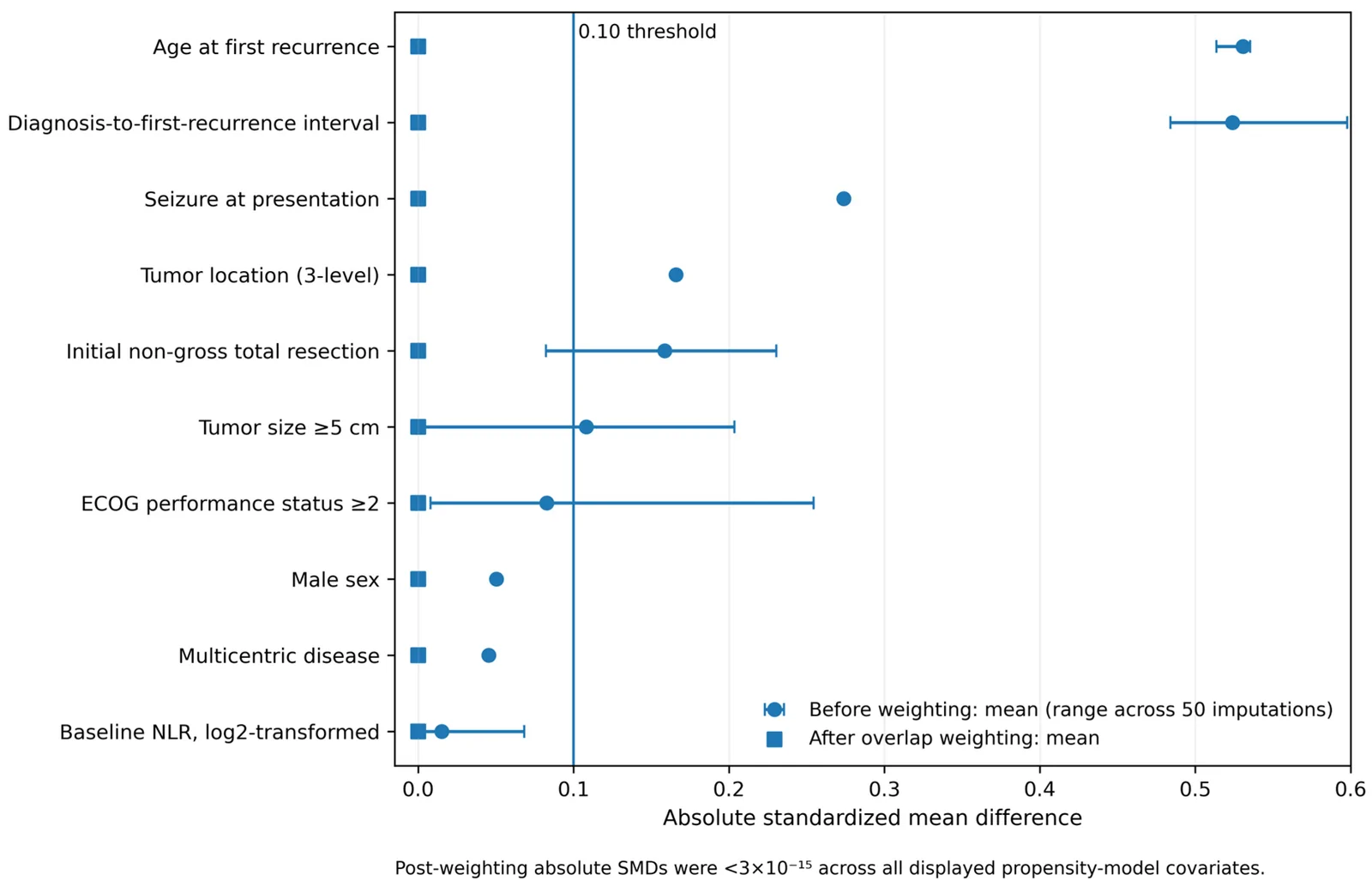
Covariate balance before and after pretreatment-only propensity-score overlap weighting. Absolute standardized mean differences are shown for the covariates included in the propensity-score model. Circles indicate the mean absolute standardized mean difference before weighting, with the range across the 50 imputed datasets; squares indicate the mean value after overlap weighting. The vertical line indicates the 0.10 threshold. For the three-level tumor-location variable, the maximum absolute standardized mean difference across categories is shown. Abbreviations: ECOG, Eastern Cooperative Oncology Group; NLR, neutrophil-to-lymphocyte ratio.

Before weighting in the multiply imputed outcome-evaluable cohort, the largest imbalances in pretreatment characteristics were observed for age at first recurrence and the interval from diagnosis to first recurrence, with mean absolute SMDs of 0.531 and 0.524, respectively, followed by seizure at presentation (0.274), tumor location (0.166), and initial non-gross total resection (0.159). After overlap weighting, absolute SMDs for all covariates included in the propensity-score model were below 0.001 in every completed dataset, consistent with the exact mean-balance property of overlap weighting for covariates entered directly into the propensity-score model (Figure 3 and Supplementary Table S10).

Propensity-score model coefficients, functional-form assessments, and weighting diagnostics are reported in Supplementary Table S11. Only the interval from diagnosis to first recurrence was significantly associated with strategy assignment (OR 0.76 per 6-month increase, 95% CI 0.62 to 0.92, *p* = 0.006). No departure from linearity was identified for any continuous covariate. The mean empirical common-support interval spanned propensity scores of 0.336 to 0.879, with a mean of 19.0 patients per completed dataset falling outside this interval; no patient had a propensity score above 0.95, and a mean of 2.1 patients in the non-bevacizumab group had a propensity score below 0.05 (Supplementary Figure S2). The mean group-specific effective sample sizes were 90.4 in the bevacizumab-containing group and 43.5 in the non-bevacizumab group, and the mean overall effective sample size was 117.4.

### 3.5. Absolute Survival Estimates

Overlap-weighted absolute estimates are reported in Supplementary Table S12. Estimated post-recurrence OS was lower with the bevacizumab-containing strategy at all four time points, with absolute differences ranging from −19.2 to −12.2 percentage points. At 18 months, the 95% CI for the difference excluded zero (23.6% versus 42.7%; difference −19.2 percentage points, 95% CI −36.1 to −0.7). Estimated prPFS was higher with the bevacizumab-containing strategy at all four time points, with absolute differences ranging from 4.3 to 13.0 percentage points; all corresponding confidence intervals included zero.

RMST differences were negative for post-recurrence OS and positive for prPFS at all four restriction horizons. At 24 months, the RMST difference was −2.75 months for post-recurrence OS (95% CI, −5.92 to 0.61) and 1.69 months for prPFS (95% CI, −1.15 to 4.55) (Supplementary Table S13). Confidence intervals for these exploratory absolute-effect analyses were not adjusted for multiple comparisons across time points or restriction horizons; the 24-month survival-probability estimates reported above should additionally be interpreted cautiously because of the limited late risk set.

### 3.6. Components of prPFS and Competing-Risk Analysis

The components of prPFS were distributed differently between the treatment strategies (Supplementary Table S14). Among the 113 patients receiving a bevacizumab-containing strategy, 23 (20.4%) had a documented second progression, 73 (64.6%) died before a documented second progression, and 17 (15.0%) were censored without either event. Among the 50 patients receiving a non-bevacizumab strategy, 33 (66.0%) had a documented second progression, 16 (32.0%) died before a documented second progression, and one (2.0%) was censored without either event.

In the overlap-weighted competing-risk analysis, the cumulative incidence of documented second progression was lower with the bevacizumab-containing strategy at all four time points, and the confidence intervals for the between-strategy differences excluded zero throughout. At 24 months, the cumulative incidence was 20.2% (95% CI, 11.6 to 29.0) with the bevacizumab-containing strategy and 55.3% (95% CI, 40.1 to 69.6) with the non-bevacizumab strategy, corresponding to a difference of −35.1 percentage points (95% CI, −52.0 to −17.7). In contrast, the cumulative incidence of death before documented second progression was higher with the bevacizumab-containing strategy at all four time points; the confidence interval for the difference included zero at 6 months but excluded zero from 12 months onward. At 24 months, the corresponding estimates were 65.5% (95% CI, 54.3 to 77.1) and 34.7% (95% CI, 21.5 to 49.9), a difference of 30.8 percentage points (95% CI, 11.8 to 48.6) (Supplementary Figure S3 and Table S15). The overlap-weighted cause-specific hazard ratio was 0.29 (95% CI, 0.16 to 0.53) for documented second progression and 1.65 (95% CI, 0.93 to 2.93) for death before documented second progression. The composite prPFS endpoint therefore combined two components that differed in opposite directions between the strategies.

### 3.7. Baseline NLR

In complete-case pretreatment-adjusted models, higher baseline NLR was associated with both endpoints when modeled continuously (Supplementary Table S16). For post-recurrence OS, the adjusted hazard ratio was 1.49 per doubling of NLR (95% CI, 1.20 to 1.85); for prPFS, the corresponding estimate was 1.43 (95% CI, 1.15 to 1.76). Restricted cubic spline models showed an overall association of NLR with both post-recurrence OS (*p* = 0.003) and prPFS (*p* = 0.004), with no evidence of departure from linearity on the log2 scale (*p* = 0.285 and *p* = 0.171, respectively) (Supplementary Figure S4).

For post-recurrence OS, there was no evidence of an interaction between treatment strategy and NLR on either scale (interaction HR 0.95, 95% CI 0.86 to 1.05, *p* = 0.320 per unit; 1.14, 95% CI 0.72 to 1.80, *p* = 0.582 per doubling). For prPFS, the interaction was not evident when NLR was modeled per unit (interaction HR 1.02, 95% CI 0.92 to 1.13, *p* = 0.746) but reached nominal significance when modeled per doubling (1.64, 95% CI 1.05 to 2.57, *p* = 0.030). In the sensitivity analysis using the historical cutoff of 3.81, the treatment-strategy association with prPFS differed numerically between NLR subgroups (adjusted HR 0.52, 95% CI 0.30 to 0.92 below the cutoff and 1.29, 95% CI 0.58 to 2.88 at or above it), but the interaction test did not reach statistical significance (*p* = 0.065); the corresponding analysis for post-recurrence OS showed no evidence of interaction (*p* = 0.447). These findings were inconsistent across NLR parameterizations and were considered exploratory rather than evidence of a reproducible treatment-modifying effect.

### 3.8. Sensitivity Analyses

Sensitivity analyses are summarized in Supplementary Table S17. Twelve patients in the primary recurrent cohort had tumors reclassified from a previous grade 3 diagnosis to WHO 2021 grade 4; one lacked evaluable post-recurrence outcomes. Excluding the remaining 11 patients left 152 patients for the corresponding sensitivity analyses. In the pretreatment-only overlap-weighted analysis after multiple imputation, the estimates were 1.37 (95% CI, 0.91 to 2.07) for post-recurrence OS and 0.87 (95% CI, 0.59 to 1.29) for prPFS, compared with 1.40 (95% CI, 0.93 to 2.10) and 0.81 (95% CI, 0.55 to 1.18), respectively, in the corresponding main analysis. Restricting the bevacizumab-containing group to bevacizumab plus irinotecan regimens yielded an HR of 1.43 (95% CI, 0.84 to 2.43) for post-recurrence OS and 0.66 (95% CI, 0.41 to 1.07) for prPFS.

Excluding patients with recurrence within 6 months of diagnosis, and separately within 3 months, yielded post-recurrence OS estimates of 1.47 (95% CI, 0.76 to 2.81) and 1.37 (95% CI, 0.79 to 2.38), respectively, and prPFS estimates of 0.50 (95% CI, 0.28 to 0.89) and 0.51 (95% CI, 0.31 to 0.84), respectively. These analyses used complete-case pretreatment-adjusted Cox models restricted to patients with an observed and chronologically valid diagnosis-to-recurrence interval (*n* = 82 and *n* = 112, respectively). In the full complete-case cohort, the corresponding estimates were 1.52 (95% CI, 0.90 to 2.58) for post-recurrence OS and 0.71 (95% CI, 0.44 to 1.13) for prPFS. The confidence intervals for prPFS excluded the null in both early-recurrence exclusion analyses.

The global proportional-hazards test indicated evidence of non-proportionality for both endpoints (*p* = 0.037 for post-recurrence OS and *p* = 0.031 for prPFS). Covariate-specific tests indicated non-proportionality for tumor size in the post-recurrence OS model and for age at first recurrence and log2-transformed NLR in the prPFS model (Supplementary Table S9). In sensitivity models allowing these covariates to vary with log-transformed follow-up time, the treatment-strategy estimate was 1.58 (95% CI, 0.92 to 2.70) for post-recurrence OS and 0.61 (95% CI, 0.38 to 0.98) for prPFS. Across the sensitivity analyses reported in this section, the prPFS confidence interval excluded the null in the two early-recurrence exclusion analyses and in the time-varying covariate model, whereas all prPFS confidence intervals in the principal analyses summarized in Table 3 included the null.

E-values were calculated for the overlap-weighted estimates obtained after multiple imputation. On the approximated risk-ratio scale, the point-estimate E-value was 1.84 for post-recurrence OS and 1.58 for prPFS. Because the 95% confidence interval for each estimate included the null, the E-value for the confidence limit closest to the null was 1.00 for both endpoints.

### 3.9. Recorded Safety Events

Recorded safety events are summarized in Supplementary Table S18. Ascertainment differed between the treatment strategies. Structured records for intracranial hemorrhage were available for 113 of 114 patients (99.1%) receiving a bevacizumab-containing strategy and for 31 of 52 patients (59.6%) receiving a non-bevacizumab strategy. Proteinuria records were available for 113 of 114 patients (99.1%) and 30 of 52 patients (57.7%), respectively. Thromboembolic-event status was available for all patients in both groups. No formal between-group testing was performed because of this difference in ascertainment.

Among evaluable patients, intracranial hemorrhage was recorded in three of 113 patients (2.7%) receiving a bevacizumab-containing strategy and two of 31 patients (6.5%) receiving a non-bevacizumab strategy, and any proteinuria in 35 of 113 (31.0%) and 11 of 30 (36.7%), respectively. Any thromboembolic event was recorded in 27 of 114 patients (23.7%) and 11 of 52 patients (21.2%), respectively, including deep vein thrombosis in 12 of 114 (10.5%) and three of 52 (5.8%), pulmonary thromboembolism in 11 of 114 (9.6%) and 7 of 52 (13.5%), and both events in three of 114 (2.6%) and one of 52 (1.9%); one additional thromboembolic event in the bevacizumab-containing group was recorded without a specified type. A complication explicitly coded as bevacizumab-related was recorded in five of 113 evaluable patients (4.4%) receiving a bevacizumab-containing strategy; an equivalent attribution variable was not available for the comparator group. Because patients received heterogeneous multimodal recurrence-directed treatment, recorded events cannot be causally attributed to a single treatment component using these retrospective data. Data on treatment discontinuation due to toxicity were not available for either group.

## 4. Discussion

In this retrospective cohort of patients with recurrent glioblastoma, a bevacizumab-containing first-recurrence strategy was not associated with improved post-recurrence OS or prPFS. In the pretreatment-only overlap-weighted analysis after multiple imputation, the hazard ratio was 1.40 (95% CI, 0.93 to 2.10) for post-recurrence OS and 0.81 (95% CI, 0.55 to 1.18) for prPFS. The two components of prPFS behaved differently. The cause-specific hazard of documented second progression was lower with the bevacizumab-containing strategy (HR 0.29, 95% CI, 0.16 to 0.53), whereas the cause-specific hazard of death before documented second progression was higher, with a confidence interval that included the null (HR 1.65, 95% CI, 0.93 to 2.93). The between-strategy difference in the cumulative incidence of death before documented second progression excluded zero from 12 months onward. This divergence indicates that a lower rate of documented progression should not by itself be read as improved disease control. Higher baseline NLR was associated with poorer outcomes for both endpoints, whereas treatment-by-NLR interactions were inconsistent across parameterizations and remained exploratory.

The comparison in this study was between two multimodal first-recurrence strategies rather than between the presence and absence of a single drug. Most patients receiving a bevacizumab-containing strategy also received irinotecan, whereas local therapy was recorded in 19.3% of that group and in 90.4% of the non-bevacizumab group. Overlap weighting balanced measured pretreatment characteristics but could not separate bevacizumab from its co-interventions. The weighted contrast therefore estimates the marginal association of initiating a bevacizumab-containing versus a non-bevacizumab first-recurrence strategy in the population with overlapping measured pretreatment characteristics, rather than the isolated pharmacologic effect of bevacizumab or the effect of a dynamic treatment regimen defined by later treatment decisions. Treatment classification was fixed at first recurrence, but 30 of 52 patients (57.7%) initially assigned to the non-bevacizumab strategy subsequently received bevacizumab later in the recurrence course. This substantial crossover could attenuate a contrast attributable specifically to the timing of bevacizumab use, although the direction and magnitude of this effect cannot be determined from these observational data.

The separation in documented second progression requires cautious interpretation. One possibility is that the bevacizumab-containing strategy delayed clinically detectable progression. However, bevacizumab can reduce vascular permeability and contrast enhancement without a proportional reduction in viable tumor, creating the well-described problem of pseudoresponse [13,14]. In this study, progression was assessed during routine care without central radiological review, and corticosteroid exposure, imaging intervals, and non-enhancing progression were not available in a form that allowed retrospective adjustment. Differential ascertainment of progression between treatment strategies therefore cannot be excluded. Because documented progression is one of the two components of the composite endpoint, differential ascertainment would also affect prPFS itself. Competing events add a separate consideration. Death before documented second progression occurred much more frequently in the bevacizumab-containing group, and such deaths reduce the cumulative incidence of subsequently documented progression. This competing-event structure may therefore contribute to the large difference in progression cumulative incidence, although it does not by itself account for the lower cause-specific hazard of documented progression. The available data cannot distinguish the extent to which the observed progression pattern reflects a true delay in progression, altered radiographic ascertainment, or differences in the competing-event process.

The prPFS findings were sensitive to several analytic choices. In the complete-case analyses, exclusion of patients with recurrence within 6 months and within 3 months of diagnosis produced hazard ratios of 0.50 and 0.51, respectively, with confidence intervals that excluded the null; allowing covariates with evidence of non-proportional hazards to vary over time yielded a corresponding estimate of 0.61. These findings were directionally consistent with the principal prPFS analyses, in which hazard ratios were also below 1, but the principal confidence intervals included the null. The early-recurrence analyses involved smaller, overlapping complete-case subsets, and exclusion of early events may also change the clinical composition of the cohort and reduce the contribution of events susceptible to early diagnostic or radiographic misclassification. The time-varying covariate analysis likewise arose from the complete-case cohort rather than an independent population. Because these analyses were exploratory, were not adjusted for multiplicity, and depended on alternative restrictions or model specifications, they should be regarded as hypothesis-generating rather than as evidence that supersedes the principal multiply imputed overlap-weighted result.

Our findings should be interpreted in the context of previous randomized and real-world evidence. The randomized phase 2 BELOB trial generated an early survival signal for bevacizumab combined with lomustine, but the subsequent phase 3 EORTC 26101 trial did not demonstrate an overall survival advantage from adding bevacizumab to lomustine [11,27]. EORTC 26101 nevertheless showed longer progression-free survival with the combination (HR 0.49, 95% CI, 0.39 to 0.61) [11], whereas the principal prPFS analyses in the present study did not exclude the null. These findings are not directly comparable. EORTC 26101 randomized patients between defined systemic regimens in which both groups received lomustine and therefore directly tested the addition of bevacizumab to a common treatment backbone. In contrast, the present study compared heterogeneous multimodal first-recurrence strategies in routine practice, with large differences in local therapy, subsequent bevacizumab use in the comparator group, and progression assessed without central review. Local salvage treatments at recurrence, including repeat resection and re-irradiation, are selected for clinically distinct patients [3], and their comparative outcomes relative to systemic therapy have been examined separately in recurrent high-grade glioma [28]. Real-world studies of bevacizumab in recurrent glioblastoma have likewise reported outcomes across heterogeneous clinical-practice populations and treatment settings [15,16,29]. By anchoring treatment classification to the first documented recurrence, restricting adjustment to pretreatment characteristics, and separating documented second progression from death before progression, the present analysis addresses the association of initiating alternative first-recurrence treatment strategies rather than the efficacy of bevacizumab as an isolated drug.

Higher baseline NLR was associated with poorer post-recurrence outcomes when modeled continuously, with no evidence of departure from linearity on the log2 scale. This finding is consistent with previous reports linking systemic inflammation to prognosis in glioblastoma [30,31,32], including a study specifically evaluating patients with recurrent glioblastoma treated with bevacizumab plus irinotecan [33]. Modeling NLR continuously avoids dependence on a single dichotomizing threshold, which is relevant because reported NLR thresholds vary across studies [32], and the historical cutoff of 3.81 was retained only as a sensitivity analysis because its original selection procedure could not be independently reverified. Evidence for treatment-effect modification was not consistent across NLR parameterizations. Although the treatment-by-log2-NLR interaction reached nominal significance for prPFS, the corresponding interaction was not evident when NLR was modeled on the raw scale and did not reach statistical significance in the historical-cutoff analysis. Taken together, these findings support NLR as a prognostic marker after recurrence but do not provide reproducible evidence that it identifies patients more or less likely to benefit from a bevacizumab-containing strategy.

Several methodological features strengthen the interpretation of these findings. Treatment strategy was classified at the first documented recurrence, which approximates the treatment decision point and reduces the immortal-time bias associated with definitions based on treatment received later in the disease course. Adjustment and propensity-score estimation were restricted to characteristics recorded before strategy assignment, so that components of the recurrence-directed strategy were not treated as confounders. Missing covariate data were addressed by multiple imputation with propensity scores and overlap weights re-estimated within each completed dataset; weighted models used robust variance estimation, and absolute survival and restricted mean survival time differences were accompanied by bootstrap confidence intervals together with balance, overlap, positivity, and effective-sample-size diagnostics. Finally, separating prPFS into documented second progression and death before documented progression revealed that its two components moved in opposite directions, a pattern that would not have been apparent from the composite endpoint alone.

Several limitations remain. First, the treatment groups represented heterogeneous multimodal strategies rather than isolated drug exposures, and substantial later use of bevacizumab in the comparator group further limits attribution of the observed associations to bevacizumab itself. Although adjustment was restricted to pretreatment characteristics, important determinants of treatment selection and prognosis at recurrence were unavailable, including recurrence-specific performance status, corticosteroid dose, radiographic tumor burden, and physician treatment preference. Residual and unmeasured confounding therefore cannot be excluded; the E-value analysis likewise does not eliminate this concern. Treatment classification was anchored to the first documented recurrence to reduce immortal-time bias, but treatment components were not necessarily initiated at that time, and the interval to bevacizumab initiation varied among exposed patients. Because a comparable treatment-initiation date could not be defined across the heterogeneous non-bevacizumab strategies, residual timing-related bias cannot be fully excluded.

Second, progression was determined from routine clinical and radiological records without blinded central review. Imaging intervals were not available as a structured variable, corticosteroid exposure could not be reconstructed, and non-enhancing progression was not separately coded; differential ascertainment and bevacizumab-related imaging effects may therefore have influenced both documented progression and the composite prPFS endpoint. Molecular characterization was also incomplete. The available dataset did not permit full reconstruction of contemporary WHO 2021 histomolecular criteria, and structured MGMT and TERT results were unavailable. Multiple imputation reduced loss of information from missing covariates but depends on assumptions about the missing-data mechanism that cannot be verified from the observed data. Safety analyses were descriptive because ascertainment of hemorrhage and proteinuria differed substantially between strategies, and treatment-specific attribution was incomplete. Censoring without either event was also more frequent in the bevacizumab-containing group, and the reasons for censoring could not be distinguished from the available records. Finally, this was a single-center study with a modest sample size and a mean effective sample size of 117.4 after overlap weighting, which may limit generalizability to centers with different recurrence-treatment practices. Several interaction, competing-risk, RMST, and sensitivity analyses were exploratory and were not adjusted for multiplicity, which limits the strength of inferences from individual secondary findings.

## 5. Conclusions

In this single-center retrospective cohort of patients with recurrent glioblastoma, initiation of a bevacizumab-containing first-recurrence strategy was not associated with improved post-recurrence OS or prPFS after adjustment for measured pretreatment characteristics, multiple imputation, and propensity-score overlap weighting. Competing-risk decomposition showed a lower cumulative incidence of documented second progression with the bevacizumab-containing strategy and a higher cumulative incidence of death before documented progression, indicating that progression-based outcomes should be interpreted cautiously in this setting.

These estimates describe associations between multimodal first-recurrence treatment strategies rather than the isolated pharmacologic effect of bevacizumab. Higher baseline NLR was associated with poorer post-recurrence outcomes, but treatment-by-NLR findings were not reproducible across parameterizations and do not support NLR-guided selection of a bevacizumab-containing strategy. Future studies with standardized imaging assessment, recurrence-specific prognostic data, more complete molecular characterization, and clearly defined treatment strategies are needed to clarify the role of bevacizumab in recurrent glioblastoma.

## Figures and Tables

**Table 1:** Clinical and treatment characteristics of patients with recurrent glioblastoma according to first-recurrence treatment strategy.

Variable	Overall	Bevacizumab-Containing Strategy	Non-Bevacizumab Strategy	*p* Value	SMD
**No. of patients**	**166**	**114**	**52**		
***Characteristics at diagnosis***					
Age at diagnosis, years	53.0 (44.2–60.8)	55.0 (46.2–63.0)	48.0 (38.5–56.0)	0.001	0.598
Male sex	96/166 (57.8)	65/114 (57.0)	31/52 (59.6)	0.753	0.053
ECOG performance status at diagnosis				0.701	0.073
0–1	104/127 (81.9)	68/84 (81.0)	36/43 (83.7)		
≥2	23/127 (18.1)	16/84 (19.0)	7/43 (16.3)		
Initial surgery				0.687	0.142
Gross total resection	131/156 (84.0)	90/105 (85.7)	41/51 (80.4)		
Subtotal resection	12/156 (7.7)	7/105 (6.7)	5/51 (9.8)		
Biopsy	13/156 (8.3)	8/105 (7.6)	5/51 (9.8)		
Tumor size ≥5 cm	45/149 (30.2)	28/99 (28.3)	17/50 (34.0)	0.473	0.124
Multicentric disease	29/166 (17.5)	20/114 (17.5)	9/52 (17.3)	0.970	0.006
Tumor location				0.747	0.129
Frontal	48/166 (28.9)	32/114 (28.1)	16/52 (30.8)		
Temporal	38/166 (22.9)	28/114 (24.6)	10/52 (19.2)		
Other	80/166 (48.2)	54/114 (47.4)	26/52 (50.0)		
Seizure at presentation	30/166 (18.1)	17/114 (14.9)	13/52 (25.0)	0.117	0.254
Neurological sequelae/deficit	83/165 (50.3)	50/113 (44.2)	33/52 (63.5)	0.022	0.393
Baseline NLR	2.82 (2.02–5.05)	2.75 (2.01–5.05)	2.86 (2.17–5.01)	0.763	0.112
Baseline NLR category				0.705	0.064
<3.81	107/164 (65.2)	72/112 (64.3)	35/52 (67.3)		
≥3.81	57/164 (34.8)	40/112 (35.7)	17/52 (32.7)		
***Characteristics at first recurrence***					
Age at first recurrence, years	54.6 (46.1–61.2)	55.5 (47.1–63.9)	51.6 (43.3–57.2)	0.013	0.473
Time from diagnosis to first recurrence, months	9.6 (5.4–17.3)	9.2 (5.1–15.3)	12.0 (6.9–21.8)	0.024	0.566
***Recurrence-directed treatment components***					
Repeat surgery	62/166 (37.3)	19/114 (16.7)	43/52 (82.7)	<0.001	1.758
Re-irradiation	20/166 (12.0)	5/114 (4.4)	15/52 (28.8)	<0.001	0.696
Systemic antineoplastic therapy during the recurrence course	155/166 (93.4)	114/114 (100.0)	41/52 (78.8)	<0.001	0.733

**Abbreviations:** ECOG, Eastern Cooperative Oncology Group; IQR, interquartile range; NLR, neutrophil-to-lymphocyte ratio; SMD, standardized mean difference. **Footnote:** Values are median (IQR) or n/N (%), with denominators reflecting available observations. Treatment-strategy assignment was defined at first recurrence: patients for whom bevacizumab was recorded as part of the first-recurrence treatment strategy were classified in the bevacizumab-containing group. ECOG performance status refers to the value documented at initial diagnosis rather than at recurrence. Age at first recurrence and time from diagnosis to first recurrence were evaluable in 163 patients (114 bevacizumab-containing and 49 non-bevacizumab) after three chronologically inconsistent diagnosis-to-recurrence intervals were treated as missing. *p* values were calculated using the Mann–Whitney U test for continuous variables and the chi-square test or Fisher exact test for categorical variables. SMDs quantify between-group imbalance; for multilevel categorical variables, the maximum absolute SMD across categories is reported, and an absolute SMD of 0.10 or greater indicates potentially meaningful imbalance. The NLR category based on the cutoff of 3.81 is shown for descriptive continuity with the original analysis; continuous NLR is used in the inferential analyses. Repeat surgery and re-irradiation represent components of the first-recurrence treatment strategy and are presented descriptively rather than as pretreatment confounders. Systemic antineoplastic therapy during the recurrence course represents treatment administered at any point after first recurrence. Treatment components were not mutually exclusive and should not be summed. The interval from first recurrence to bevacizumab initiation was available only for patients receiving a bevacizumab-containing strategy and is reported in the Results.

**Table 2:** Pretreatment-adjusted Cox proportional hazards regression analyses for post-recurrence overall survival.

Variable	Univariable HR (95% CI)	*p* Value	Multivariable HR (95% CI)	*p* Value
Bevacizumab-containing first-recurrence strategy versus non-bevacizumab strategy	1.57 (1.08–2.29)	0.017	1.52 (0.90–2.58)	0.117
Age at first recurrence, per 10-year increase	1.19 (1.01–1.40)	0.033	1.21 (0.99–1.49)	0.060
ECOG performance status ≥ 2 at diagnosis	1.29 (0.79–2.10)	0.308	1.20 (0.68–2.13)	0.531
Baseline NLR, per doubling	1.28 (1.06–1.54)	0.010	1.49 (1.20–1.85)	<0.001
Time from diagnosis to first recurrence, per 6-month increase	0.94 (0.87–1.01)	0.076	0.97 (0.91–1.05)	0.476
Tumor size ≥ 5 cm	0.93 (0.62–1.40)	0.727	0.95 (0.58–1.55)	0.829
Multicentric disease	1.22 (0.78–1.92)	0.389	1.18 (0.67–2.09)	0.570
Initial non-gross total resection	1.38 (0.89–2.15)	0.150	1.95 (1.16–3.27)	0.011

**Abbreviations:** CI, confidence interval; ECOG, Eastern Cooperative Oncology Group; HR, hazard ratio; NLR, neutrophil-to-lymphocyte ratio. **Footnote:** Post-recurrence overall survival was defined as the interval from first documented recurrence or progression to death from any cause or last known follow-up. Initial non-gross total resection included biopsy and subtotal resection. Baseline NLR was modeled continuously after log2 transformation because of its right-skewed distribution; the reported hazard ratio therefore represents the relative hazard associated with a doubling of NLR. The multivariable model included only characteristics available before or at the definition of the first-recurrence treatment strategy and was performed as a complete-case analysis including 117 patients with 91 deaths. Repeat surgery and re-irradiation were not included because they represent components of the recurrence-directed treatment strategy rather than pretreatment confounders. Hazard ratios greater than 1 indicate an increased hazard of death after first recurrence. Univariable analyses used available observations for each covariate.

**Table 3:** Comparative analyses of the bevacizumab-containing first-recurrence treatment strategy.

Endpoint	Analysis	n/Events	HR (95% CI)	*p* Value
**Post-recurrence OS**	Unadjusted Cox model	163/136	1.57 (1.08–2.29)	0.017
	Pretreatment-adjusted Cox model, complete case	117/91	1.52 (0.90–2.58)	0.117
	Pretreatment-adjusted Cox model after MI	163/136	1.36 (0.88–2.09)	0.167
	Pretreatment-only overlap-weighted Cox model, complete case	117/91	1.34 (0.82–2.19)	0.235
	Pretreatment-only overlap-weighted Cox model after MI	163/136	1.40 (0.93–2.10)	0.107
**prPFS**	Unadjusted Cox model	163/145	0.87 (0.61–1.23)	0.433
	Pretreatment-adjusted Cox model, complete case	117/100	0.71 (0.44–1.13)	0.150
	Pretreatment-adjusted Cox model after MI	163/145	0.78 (0.52–1.17)	0.230
	Pretreatment-only overlap-weighted Cox model, complete case	117/100	0.68 (0.43–1.08)	0.102
	Pretreatment-only overlap-weighted Cox model after MI	163/145	0.81 (0.55–1.18)	0.267

**Abbreviations:** CI, confidence interval; HR, hazard ratio; MI, multiple imputation; OS, overall survival; prPFS, post-recurrence progression-free survival. **Footnote:** Hazard ratios compare initiation of a bevacizumab-containing first-recurrence treatment strategy with a non-bevacizumab first-recurrence strategy. The pretreatment-adjusted Cox models included treatment strategy, age at first recurrence, ECOG performance status at diagnosis, baseline NLR modeled continuously on the log2 scale, time from diagnosis to first recurrence, tumor size, multicentric disease, and initial extent of resection. The pretreatment-only propensity-score model included age at first recurrence, sex, ECOG performance status at diagnosis, continuous log2-transformed baseline NLR, time from diagnosis to first recurrence, initial non-gross total resection, tumor size, multicentric disease, tumor location, and seizure at presentation. Repeat surgery and re-irradiation were excluded from the propensity-score model because they were components of the recurrence-directed treatment strategy rather than pretreatment confounders. Neurological sequelae were not included because their temporal relationship to treatment assignment could not be established reliably. Multiple imputation by chained equations was performed in the 163 patients with evaluable post-recurrence outcomes using 50 imputed datasets; survival outcomes were not imputed. Propensity scores and overlap weights were re-estimated separately within each imputed dataset. Overlap weights were calculated as 1 minus the propensity score for patients receiving a bevacizumab-containing strategy and as the propensity score for patients receiving a non-bevacizumab strategy. Estimates from multiply imputed analyses were combined using Rubin’s rules. Robust sandwich variance estimates were used for overlap-weighted Cox models. Tied event times were handled using the Breslow method. Hazard ratios greater than 1 indicate a higher outcome hazard and hazard ratios less than 1 a lower outcome hazard with the bevacizumab-containing strategy.

## Data Availability

The data presented in this study are available on request from the corresponding author due to ethical and privacy restrictions.

## Supplementary Materials

The following supporting information can be downloaded at: https://www.mdpi.com/article/10.3390/cancers18162665/s1, Figure S1: Post-recurrence progression-free survival according to first-recurrence treatment strategy; Figure S2: Distribution of estimated propensity scores according to first-recurrence treatment strategy; Figure S3: Competing-risk cumulative incidence according to first-recurrence treatment strategy; Figure S4: Continuous association between baseline neutrophil-to-lymphocyte ratio and post-recurrence outcomes; Table S1: Cohort derivation; Table S2: Distribution of tumors before and after WHO 2021 integrated reclassification; Table S3: Availability of structured molecular and pathological data and reconciliation with the WHO 2021 integrated diagnosis; Table S4: Comparison of patients included in the primary recurrent cohort and those without documented first recurrence; Table S5: Missing-data pattern and handling; Table S6: Treatment patterns at first recurrence; Table S7: Subsequent treatment lines and attrition after second and later progressions; Table S8: Cox regression analyses for post-recurrence progression-free survival; Table S9: Proportional-hazard diagnostics and time-varying covariate sensitivity analyses; Table S10: Covariate balance before and after overlap weighting; Table S11: Propensity-score model specification, functional-form assessment, and weighting diagnostics; Table S12: Overlap-weighted absolute survival estimates; Table S13: Overlap-weighted restricted mean survival time; Table S14: Components of post-recurrence progression-free survival events; Table S15: Overlap-weighted competing-risk analysis; Table S16: Continuous, nonlinear, and interaction analyses of baseline neutrophil-to-lymphocyte ratio; Table S17: Sensitivity analyses; Table S18: Recorded safety events. The completed STROBE checklist is provided as a separate supplementary file.
