# Supplementary material for: Post-Recurrence Outcomes Associated with a Bevacizumab-Containing First-Recurrence Strategy in Glioblastoma: A Propensity-Weighted Single-Center Cohort Study with Competing-Risk Analysis
Source: Cancers (Basel). 2026 Aug 18;18(16):2665. doi: 10.3390/cancers18162665 (PMC13510414; doi:10.3390/cancers18162665)
Supplement: Supplementary file 1 [file cancers-18-02665-s001.zip › STROBE_checklist.pdf]

STROBE Statement Checklist for Cohort Studies

Post-Recurrence Outcomes Associated with a Bevacizumab-Containing First-Recurrence Strategy in Glioblastoma: A Propensity-Weighted Single-Center Cohort Study with Competing-Risk Analysis

STROBE checklist for cohort studies, mapped to the corresponding sections of the manuscript.

STROBE section (item no.)	Recommendation	Location in manuscript
Title and abstract (1a)	Indicate the study's design in the title or abstract	Title; Abstract (Methods)
Title and abstract (1b)	Provide a balanced summary of what was done and found	Abstract
Introduction – Background/rationale (2)	Explain the scientific background and rationale	Introduction, paragraphs 1–4
Introduction – Objectives (3)	State specific objectives, including any prespecified hypotheses	Introduction, paragraph 5. The study was not conducted under a predefined analysis protocol; objectives are stated as association analyses, and interaction, competing-risk, restricted mean survival time, subgroup, and sensitivity analyses are identified as exploratory.
Methods – Study design (4)	Present key elements of study design early	Section 2.1
Methods – Setting (5)	Describe setting, locations, and relevant dates, including recruitment and follow-up periods	Section 2.1 (single center; January 2010 to December 2022; follow-up updated through 15 June 2023)
Methods – Participants (6)	Give eligibility criteria and sources and methods of selection	Section 2.1; Figure 1; Supplementary Table S1
Methods – Variables (7)	Clearly define outcomes, exposures, predictors, potential confounders, and effect modifiers	Sections 2.2, 2.3, and 2.4
Methods – Data sources/measurement (8)	Give sources of data and details of assessment for each variable	Sections 2.1 to 2.4; Supplementary Tables S3 and S5
Methods – Bias (9)	Describe efforts to address potential sources of bias	Section 2.2 (immortal-time bias); Section 2.3 (progression ascertainment); Section 2.4 (confounding by indication, restriction of adjustment to pretreatment characteristics, overlap weighting, E-values)

STROBE section (item no.)	Recommendation	Location in manuscript
Methods – Study size (10)	Explain how the study size was arrived at	Section 2.1; Figure 1 (all eligible consecutive patients)
Methods – Quantitative variables (11)	Explain how quantitative variables were handled, including groupings	Section 2.4. Baseline NLR was modeled continuously on the raw and log2 scales with restricted cubic splines; the historical cutoff of 3.81 was retained only for sensitivity analysis.
Methods – Statistical methods (12a)	Describe all statistical methods, including those used to control for confounding	Section 2.4; Supplementary Tables S10 and S11
Methods – Statistical methods (12b)	Describe any methods used to examine subgroups and interactions	Section 2.4; Section 3.7; Supplementary Table S16
Methods – Statistical methods (12c)	Explain how missing data were addressed	Section 2.4 (multiple imputation by chained equations, 50 completed datasets); Supplementary Table S5
Methods – Statistical methods (12d)	If applicable, explain how loss to follow-up was addressed	Sections 2.3 and 3.6; Supplementary Table S14
Methods – Statistical methods (12e)	Describe any sensitivity analyses	Section 2.4; Section 3.8; Supplementary Tables S9 and S17
Results – Participants (13a–c)	Report numbers at each stage, reasons for non-participation, and a flow diagram	Section 2.1; Figure 1; Supplementary Tables S1 and S4
Results – Descriptive data (14a–c)	Characteristics of participants, missing data, and follow-up time	Sections 3.1 and 3.2; Table 1; Supplementary Tables S5 and S6. The follow-up cut-off date is given in Section 2.1; a summary follow-up duration is not reported.
Results – Outcome data (15)	Report numbers of outcome events or summary measures over time	Sections 3.3 and 3.6; Tables 2 and 3; Supplementary Tables S14 and S15
Results – Main results (16a)	Give unadjusted estimates and, if applicable, confounder-adjusted estimates and their precision (e.g., 95% confidence interval). Make clear which confounders were adjusted for and why they were included	Sections 3.3 and 3.4; Tables 2 and 3; Figures 2 and 3; Supplementary Tables S8, S10, and S11
Results – Main results (16b)	Report category boundaries when continuous variables were categorized	Section 2.4 and Section 3.7 (historical NLR cutoff of 3.81 used only in sensitivity analysis); Table 1
Results – Main results (16c)	If relevant, consider translating estimates of relative risk into absolute risk for a meaningful time period	Section 3.5. Overlap-weighted absolute survival probabilities at 6, 12, 18, and 24 months, between-strategy differences in

STROBE section (item no.)	Recommendation	Location in manuscript
		percentage points, and restricted mean survival time differences are reported; Supplementary Tables S12 and S13. Absolute competing-risk cumulative incidences are reported in Section 3.6 and Supplementary Table S15.
Results – Other analyses (17)	Report other analyses (subgroups, interactions, sensitivity analyses)	Sections 3.6, 3.7, and 3.8; Supplementary Tables S9, S15, S16, and S17
Discussion – Key results (18)	Summarize key results with reference to study objectives	Section 4, paragraph 1
Discussion – Limitations (19)	Discuss limitations, sources of bias, and their direction and magnitude	Section 4, final two paragraphs
Discussion – Interpretation (20)	Give a cautious overall interpretation considering objectives and other evidence	Section 4, paragraphs 2 to 6; Section 5
Discussion – Generalisability (21)	Discuss the generalisability of the results	Section 4, final paragraph; Section 5
Other information – Funding (22)	Give the source of funding and the role of funders	Funding statement

STROBE, Strengthening the Reporting of Observational Studies in Epidemiology. This checklist maps each STROBE item for cohort studies to the corresponding section, table, or figure in the revised manuscript. Section and table references are provided because pagination may change during manuscript processing.
