# Supplementary material for: Post-Recurrence Outcomes Associated with a Bevacizumab-Containing First-Recurrence Strategy in Glioblastoma: A Propensity-Weighted Single-Center Cohort Study with Competing-Risk Analysis
Source: Cancers (Basel). 2026 Aug 18;18(16):2665. doi: 10.3390/cancers18162665 (PMC13510414; doi:10.3390/cancers18162665)
Supplement: Supplementary file 1 [file cancers-18-02665-s001.zip › supplementary figures and tables.pdf]

Supplementary Materials

Cancers

Post-Recurrence Outcomes Associated with a Bevacizumab-Containing First-Recurrence Strategy in Glioblastoma: A Propensity-Weighted Single-Center Cohort Study with Competing-Risk Analysis

Enes Yeşilbaş [1,*] and Sema Sezgin Göksu [2]

[1] Department of Medical Oncology, Ankara Etlik City Hospital, Ankara 06010, Türkiye
[2] Department of Medical Oncology, Faculty of Medicine, Akdeniz University,

Antalya 07070, Türkiye; semasezgingoksu@gmail.com
* Correspondence: yesilbas126@gmail.com; Tel.: +90-532-179-5590

Supplementary Figures

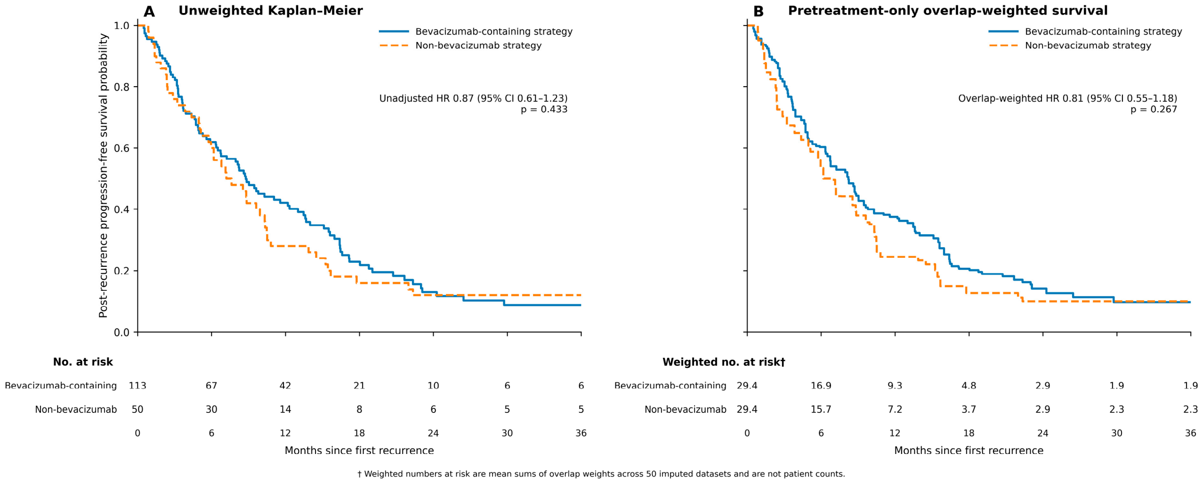

Figure S1. Post-recurrence progression-free survival according to first-recurrence treatment strategy. Panel A shows unweighted Kaplan–Meier curves in the outcome-evaluable cohort (n = 163), together with the unadjusted Cox proportional hazards estimate. Panel B shows survival curves after pretreatment-only propensity-score overlap weighting, averaged across 50 multiply imputed datasets, together with the corresponding pooled overlap-weighted Cox estimate. Propensity scores were estimated separately within each imputed dataset using the pretreatment-only model. Numbers at risk in Panel A represent observed patient counts. Weighted numbers at risk in Panel B represent mean sums of overlap weights across the 50 imputed datasets and should not be interpreted as patient counts. The x-axis was truncated at

36 months for graphical presentation only; all available follow-up was retained in the Cox analyses. Abbreviations: CI, confidence interval; HR, hazard ratio; prPFS, post-recurrence progression-free survival.

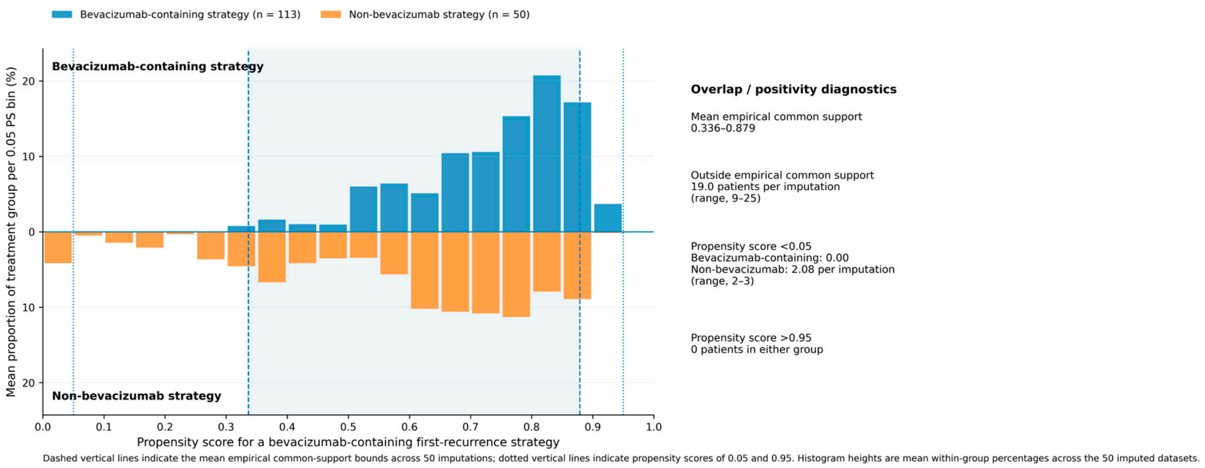

Figure S2. Distribution of estimated propensity scores according to first-recurrence treatment strategy. Mirrored histograms show the distribution of propensity scores for initiation of a bevacizumab-containing first-recurrence treatment strategy in the outcome-evaluable cohort. Histogram heights represent the mean within-group percentage of patients in each 0.05-wide propensity-score interval across 50 multiply imputed datasets. Propensity scores were estimated separately within each imputed dataset using the pretreatment-only logistic propensity-score model. The shaded region represents the mean empirical common-support interval across imputations, with dashed vertical lines indicating its mean lower and upper bounds. Dotted vertical lines indicate propensity scores of 0.05 and 0.95. The mean empirical common-support interval was 0.336 to 0.879; its lower and upper bounds ranged from 0.295 to 0.360 and from 0.858 to 0.933, respectively, across imputations. A mean of 19.0 patients per imputed dataset were outside the strict empirical common-support interval. No patient in the bevacizumab-containing strategy group had a propensity score below 0.05, whereas a mean of 2.08 patients per imputed dataset in the non-bevacizumab strategy group had propensity scores below 0.05. No estimated propensity score exceeded 0.95 in either group. Abbreviations: PS, propensity score.

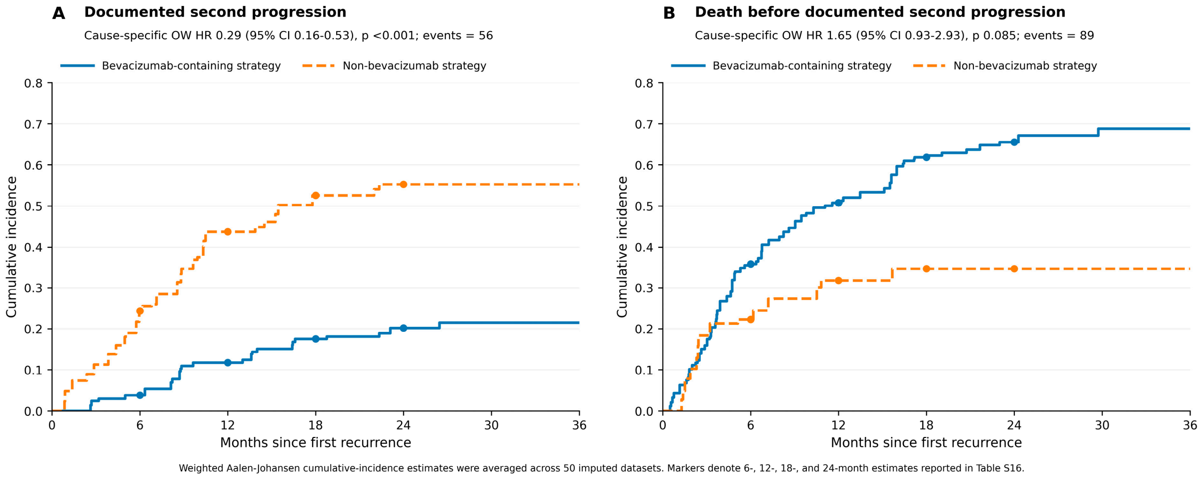

Figure S3. Competing-risk cumulative incidence according to first-recurrence treatment strategy. Weighted Aalen–Johansen cumulative-incidence functions were estimated separately within each of 50 multiply imputed datasets using pretreatment-only propensity-score overlap weights and were subsequently averaged across imputations. Panel A shows the cumulative incidence of documented second progression, treating death before documented second progression as the competing event. Panel B shows the cumulative incidence of death before documented second progression, treating documented second progression as the competing event. Solid and dashed curves represent bevacizumab-containing and non-bevacizumab first-recurrence treatment strategies, respectively. Markers denote the 6-, 12-, 18-, and 24-month estimates reported in Table S15. Cause-specific overlap-weighted Cox hazard ratios compare the bevacizumab-containing strategy with the non-bevacizumab strategy. Among 163 outcome-evaluable patients, 56 experienced documented second progression, 89 died before documented second progression, and 18 were censored without either event. Abbreviations: CI, confidence interval; HR, hazard ratio; OW, overlap weighting.

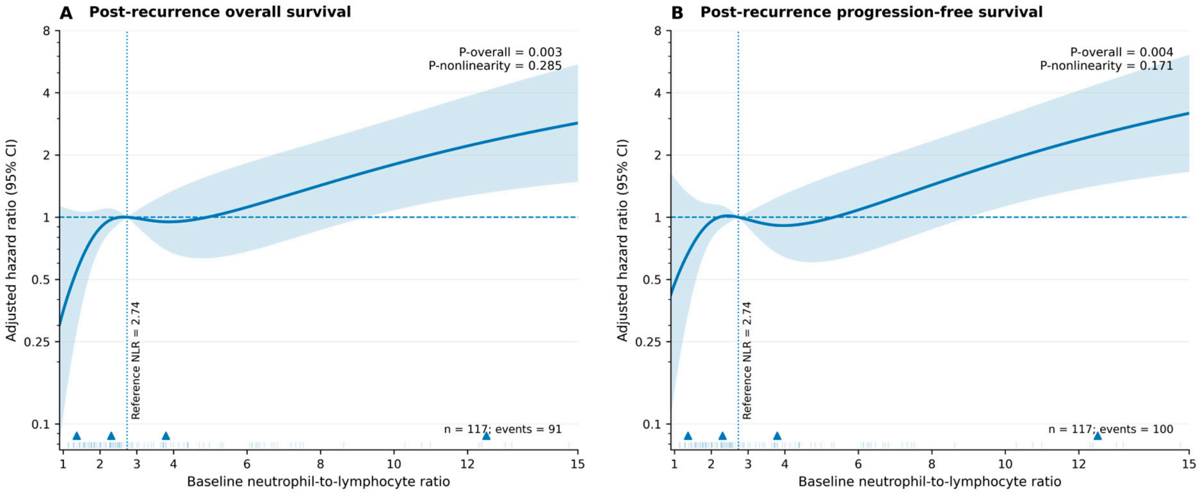

Figure S4. Continuous association between baseline neutrophil-to-lymphocyte ratio and post-recurrence outcomes. Adjusted hazard ratios and 95% confidence intervals were estimated using restricted cubic splines for baseline neutrophil-to-lymphocyte ratio in complete-case pretreatment-adjusted Cox proportional hazards models (n = 117). Panel A shows post-recurrence overall survival and Panel B shows post-recurrence progression-free survival. Baseline NLR was modeled on the log2 scale using four knots placed at the 5th, 35th, 65th, and 95th percentiles, corresponding to raw NLR values of 1.37, 2.31, 3.79, and 12.51. Hazard ratios were normalized to the cohort median NLR of 2.74. Models were adjusted for first-recurrence treatment strategy, age at first recurrence, ECOG performance status ≥2 at diagnosis, time from diagnosis to first recurrence, tumor size ≥5 cm, multicentric disease, and initial non-gross total resection. P-overall values were obtained from likelihood-ratio tests comparing the spline model with the corresponding model excluding NLR, whereas P-nonlinearity values compared the spline model with a model containing linear log2-transformed NLR. Rug marks show the observed NLR distribution and triangles indicate spline-knot locations. For graphical presentation, the x-axis was restricted to NLR values from 0.9 to 15; three observations above 15 were retained in model estimation. Abbreviations: CI, confidence interval; ECOG, Eastern Cooperative Oncology Group; HR, hazard ratio; NLR, neutrophil-to-lymphocyte ratio; OS, overall survival; prPFS, post-recurrence progression-free survival.

Supplementary Tables

Table S1. Cohort derivation for the primary recurrent WHO 2021 glioblastoma, IDH-wildtype analysis cohort.

Cohort derivation step	n	%
Patients with histopathologically confirmed high-grade glioma screened	350	100.0
Excluded because of incomplete clinical or radiological documentation	26	7.4
Final high-grade glioma cohort	324	92.6
WHO 2021 glioblastoma, IDH-wildtype	266	82.1
Excluded because assigned to non-glioblastoma integrated diagnostic categories	58	17.9
WHO 2021 glioblastoma, IDH-wildtype without documented first recurrence/progression	100	37.6
Primary recurrent WHO 2021 glioblastoma, IDH-wildtype cohort	166	62.4
Bevacizumab-containing first-recurrence strategy	114	68.7
Non-bevacizumab first-recurrence strategy	52	31.3

Abbreviations: IDH, isocitrate dehydrogenase; WHO, World Health Organization.
Footnote: Percentages for the screened and final high-grade glioma cohorts were calculated using the screened population as the denominator. Percentages for WHO 2021 diagnostic groups were calculated using the final high-grade glioma cohort as the denominator. Percentages for patients with and without documented first recurrence/progression were calculated using the WHO 2021 glioblastoma, IDH-wildtype cohort as the denominator. Percentages for first-recurrence treatment strategies were calculated using the primary recurrent WHO 2021 glioblastoma, IDH-wildtype cohort as the denominator. The bevacizumab-containing strategy was defined as receipt of any bevacizumab-containing regimen as part of the first-recurrence treatment strategy.

Table S2. Distribution of tumors before and after WHO 2021 integrated reclassification

Panel A. *Reclassification from WHO 2016 histologic subtype to WHO 2021 integrated diagnosis.*

WHO 2016 histologic subtype	Astrocytoma, IDH-mutant	Oligodendroglioma, IDH-mutant and 1p/19q-codeleted	Glioblastoma, IDH-wildtype	Total
Glioblastoma	26 (9.4)	0 (0.0)	252 (90.6)	278 (100.0)
Anaplastic astrocytoma	9 (39.1)	0 (0.0)	14 (60.9)	23 (100.0)
Anaplastic oligodendroglioma	0 (0.0)	20 (100.0)	0 (0.0)	20 (100.0)
Anaplastic oligoastrocytoma	3 (100.0)	0 (0.0)	0 (0.0)	3 (100.0)
**Total**	**38 (11.7)**	**20 (6.2)**	**266 (82.1)**	**324 (100.0)**

Panel B. *Distribution of tumor grade before and after WHO 2021 reclassification.*

Previous WHO grade	WHO 2021 grade 3	WHO 2021 grade 4	Total
Grade 3	32 (69.6)	14 (30.4)	46 (100.0)
Grade 4	0 (0.0)	278 (100.0)	278 (100.0)
**Total**	**32 (9.9)**	**292 (90.1)**	**324 (100.0)**

Abbreviations: IDH, isocitrate dehydrogenase; WHO, World Health Organization.

Footnote: Values are n (%). Percentages in the body of each panel are row percentages, whereas percentages in the total row are calculated using the overall high-grade glioma cohort as the denominator (n = 324). WHO 2021 integrated diagnostic categories reflect the final integrated pathological diagnosis recorded in the clinical dataset together with the available structured molecular information. Fourteen tumors previously classified as grade 3 anaplastic astrocytoma were assigned to the glioblastoma, IDH-wildtype category and therefore classified as grade 4; all 14 had an IDH1 wild-type/not-mutant result recorded in the structured dataset. Twelve of these 14 patients were included in the primary recurrent cohort. The structured dataset did not capture the method of IDH assessment or the complete histological and molecular evidence required to reconstruct complete WHO 2021 histomolecular confirmation retrospectively. Sensitivity analyses excluding the 12 grade 3-to-grade 4 reclassified patients from the recurrent cohort are presented separately.

Table S3. Availability of structured molecular and pathological data and reconciliation with the WHO 2021 integrated diagnosis in the final high-grade glioma cohort

Panel A. *Availability of structured molecular and pathological markers.*

Marker	Evaluable/tested, n (%)	Recorded result	Not available/missing, n (%)	Comment
IDH1 mutation status	168 (51.9)	Mutant: 45 (26.8); wild-type/not-mutant: 123 (73.2)	156 (48.1)	Direct result recorded in the structured IDH1 field
1p/19q deletion status	38 (11.7)	Deletion-coded: 22 (57.9); non-deletion-coded: 16 (42.1)	286 (88.3)	Direct result recorded in the structured 1p/19q field
p53 status	196 (60.5)	Positive/mutant-coded: 164 (83.7); negative/wild-coded: 32 (16.3)	128 (39.5)	Supportive pathological marker; not used alone to establish WHO 2021 integrated diagnosis
Ki-67 proliferation index	224 (69.1)	Median: 20% (IQR, 10–30); range, 1–100	100 (30.9)	Proliferation marker
MGMT promoter methylation	0 (0.0)	Not available	324 (100.0)	Not captured in the structured dataset
TERT promoter alteration	0 (0.0)	Not available	324 (100.0)	Not captured in the structured dataset

Panel B. *Reconciliation of structured IDH1 status with WHO 2021 integrated diagnosis.*

WHO 2021 integrated diagnosis	Total, n	Structured IDH1 mutant	Structured IDH1 wild-type/not-mutant	Structured IDH1 result unavailable
Astrocytoma, IDH-mutant	38	33 (86.8)	0 (0.0)	5 (13.2)
Oligodendroglioma, IDH-mutant and 1p/19q-codeleted	20	12 (60.0)	0 (0.0)	8 (40.0)
Glioblastoma, IDH-wildtype	266	0 (0.0)	123 (46.2)	143 (53.8)
**Total**	**324**	**45 (13.9)**	**123 (38.0)**	**156 (48.1)**

Abbreviations: IDH, isocitrate dehydrogenase; IQR, interquartile range; MGMT, O6-methylguanine-DNA methyltransferase; TERT, telomerase reverse transcriptase; WHO, World Health Organization.

Footnote: Values are n (%) unless otherwise specified. Percentages for marker availability and missingness were calculated using the final high-grade glioma cohort as the denominator (n = 324), whereas percentages for marker results in Panel A were calculated among patients with an available structured result. Panel B distinguishes the final WHO 2021 integrated diagnostic category from the directly recorded structured IDH1 result. Among the 58 tumors assigned to an IDH-mutant WHO 2021 integrated diagnosis, 45 had a directly recorded IDH1-mutant result and 13 had no structured IDH1 result; the latter comprised 5 astrocytomas and

8 oligodendrogliomas. Among the 266 tumors assigned to the glioblastoma, IDH-wildtype category, 123 had a directly recorded IDH1 wild-type/not-mutant result and 143 had no structured IDH1 result. Accordingly, integrated diagnostic category counts should not be interpreted as counts of directly documented IDH1 test results. The direct structured 1p/19q field contained 22 deletion-coded and 16 non-deletion-coded results among 38 evaluable cases and is reported without post hoc recoding to match the integrated diagnostic categories. IDH assessment method, histological grade 4 features, EGFR amplification, chromosome 7 gain/chromosome 10 loss, and other elements required to reconstruct complete histomolecular confirmation were not available as structured variables; therefore, complete WHO 2021 histomolecular confirmation could not be reliably reconstructed retrospectively.

Table S4. Comparison of patients with a WHO 2021 integrated diagnosis of glioblastoma, IDH-wildtype included in the primary recurrent cohort and those without documented first recurrence/progression

Variable	Primary recurrent cohort, n = 166	No documented first recurrence/progression, n = 100	p value	SMD
Age at diagnosis, years	53.0 (44.2–60.8)	64.5 (55.8–70.0)	<0.001	0.849
Male sex	96/166 (57.8)	58/100 (58.0)	0.978	0.003
ECOG performance status ≥2 at diagnosis	23/127 (18.1)	30/96 (31.2)	0.022	0.308
**Initial surgery**			**0.196**	**0.209**
Gross total resection	131/156 (84.0)	80/100 (80.0)		
Subtotal resection	12/156 (7.7)	5/100 (5.0)		
Biopsy	13/156 (8.3)	15/100 (15.0)		
Tumor size ≥5 cm	45/149 (30.2)	27/100 (27.0)	0.585	0.071
Multicentric disease	29/166 (17.5)	18/100 (18.0)	0.913	0.014
**Tumor location**			**0.834**	**0.076**
Frontal	**48/166 (28.9)**	27/100 (27.0)		
Temporal	38/166 (22.9)	21/100 (21.0)		
Other	**80/166 (48.2)**	52/100 (52.0)		
Seizure at presentation	30/166 (18.1)	14/100 (14.0)	0.387	0.111
Neurological sequelae/deficit	**83/165 (50.3)**	41/100 (41.0)	**0.141**	**0.188**
Ki-67 proliferation index, %	20.0 (10.0–35.0)	20.0 (10.0–30.0)	0.710	0.280
Baseline NLR	2.8 (2.0–5.1)	3.6 (2.0–7.7)	0.027	0.172
Baseline NLR ≥3.81	57/164 (34.8)	48/100 (48.0)	0.033	0.271
Initial radiotherapy + temozolomide	153/166 (92.2)	90/99 (90.9)	0.719	0.045
Adjuvant temozolomide ≥6 cycles	**107/133 (80.5)**	47/73 (64.4)	**0.011**	**0.365**
Deceased at last follow-up	136/163 (83.4)	74/91 (81.3)	0.653	0.058

Abbreviations: ECOG, Eastern Cooperative Oncology Group; IDH, isocitrate dehydrogenase; IQR, interquartile range; NLR, neutrophil-to-lymphocyte ratio; SMD, standardized mean difference; WHO, World Health Organization.

Footnote: Values are median (IQR) or n/N (%). This table compares patients assigned to the WHO 2021 integrated glioblastoma, IDH-wildtype diagnostic category who were included in the primary recurrent analysis cohort with patients in the same integrated diagnostic category who had no documented first recurrence/progression and were therefore not eligible for the first-recurrence comparative-effectiveness analyses. ECOG performance status refers to the value documented at initial diagnosis. Baseline NLR refers to the value obtained at initial treatment. The NLR cutoff of 3.81 is retained for descriptive continuity only; continuous NLR is used in the revised primary modeling strategy. P values were calculated using the Mann–Whitney U test for continuous variables and the chi-square or Fisher exact test for categorical variables. SMDs quantify between-group imbalance; for multilevel categorical variables, the maximum absolute SMD across categories is reported. An absolute SMD of 0.10 or greater

was considered indicative of meaningful imbalance. Denominators reflect available observations for each variable.

Table S5. Missing-data pattern and handling in the revised complete-case and multiple-imputation analyses

Variable	Available before imputation, n/N (%)	Missing before imputation, n/N (%)	Imputed in MI analyses	Included in imputation model	Role in revised analyses
Age at diagnosis	166/166 (100.0)	0/166 (0.0)	No	Yes	Auxiliary predictor; used to derive age at first recurrence
Age at first recurrence	**163/166 (98.2)**	**3/166 (1.8)**	**Not separately; derived after imputation of recurrence interval**	No, derived variable	Cox and PS models
Sex	166/166 (100.0)	0/166 (0.0)	No	Yes	PS model
ECOG performance status ≥2 at diagnosis	127/166 (76.5)	**39/166 (23.5)**	**Yes**	Yes	Cox and PS models
Baseline NLR, log2-transformed	164/166 (98.8)	2/166 (1.2)	**Yes**	Yes	Cox and PS models
Time from diagnosis to first recurrence	**163/166 (98.2)**	**3/166 (1.8)**	**Yes**	Yes	Cox and PS models; derivation of age at recurrence
Initial non-gross total resection	156/166 (94.0)	10/166 (6.0)	**Yes**	Yes	Cox and PS models
Tumor size ≥5 cm	149/166 (89.8)	17/166 (10.2)	**Yes**	Yes	Cox and PS models
Multicentric disease	166/166 (100.0)	0/166 (0.0)	No	Yes	Cox and PS models
Tumor location	166/166 (100.0)	0/166 (0.0)	No	Yes	PS model
Seizure at presentation	166/166 (100.0)	0/166 (0.0)	No	Yes	PS model
Bevacizumab-containing first-recurrence strategy	166/166 (100.0)	0/166 (0.0)	No	Yes	Exposure
Post-recurrence OS status/time	163/166 (98.2)	3/166 (1.8)	**No**	Yes, as outcome information	Outcome
prPFS status/time	163/166 (98.2)	3/166 (1.8)	**No**	Yes, as outcome information	Outcome
**Outcome-evaluable analysis population**	**163/166 (98.2)**	**3/166 (1.8)**	Not applicable	Not applicable	MI and survival analyses
**Complete cases among outcome-evaluable patients**	**117/163 (71.8)**	**46/163 (28.2)**	Not applicable	Not applicable	Complete-case analyses

Abbreviations: ECOG, Eastern Cooperative Oncology Group; MI, multiple imputation; NLR, neutrophil-to-lymphocyte ratio; OS, overall survival; prPFS, post-recurrence progression-free survival; PS, propensity score.

Footnote: Values are n/N (%). Original missingness was assessed in the primary recurrent cohort (n = 166). Three patients did not have evaluable post-recurrence survival outcomes and were excluded before survival modeling and multiple imputation; survival outcomes were not imputed. Multiple imputation by chained equations was performed in the 163 outcome-evaluable patients using 50 imputed datasets. Continuous diagnosis-to-recurrence time and log2-transformed baseline NLR were imputed using predictive mean matching, whereas ECOG performance status of 2 or higher, initial non-gross total resection, and tumor size of 5 cm or larger were imputed using binary logistic chained-equation models. Bootstrap perturbation was used within the chained-equation procedure to incorporate imputation-model uncertainty. Age at first recurrence was not separately imputed; it was recalculated from age at diagnosis and the observed or imputed diagnosis-to-recurrence interval. Treatment strategy, fully observed pretreatment covariates, survival-event indicators, and Nelson-Aalen cumulative

hazard estimates were included as predictors where applicable but were not themselves imputed. Propensity scores and overlap weights were re-estimated independently within each imputed dataset. Regression coefficients and their variances were combined across imputations using Rubin's rules. The revised propensity-score model was restricted to pretreatment characteristics; relapse surgery and re-irradiation/CyberKnife were not included as propensity-score covariates.

Table S6. Treatment patterns at first recurrence according to first-recurrence treatment strategy in the primary recurrent WHO 2021 glioblastoma, IDH-wildtype cohort

Treatment pattern	Overall, n = 166	Bevacizumab-containing strategy, n = 114	Non-bevacizumab strategy, n = 52
Non-mutually exclusive treatment components			
Any bevacizumab-containing regimen	114 (68.7)	114 (100.0)	0 (0.0)
Any irinotecan-containing regimen	110 (66.3)	109 (95.6)	1 (1.9)
Any temozolomide-containing regimen	25 (15.1)	0 (0.0)	25 (48.1)
Any systemic drug-containing regimen	140 (84.3)	114 (100.0)	26 (50.0)
Re-operation at first recurrence	62 (37.3)	19 (16.7)	43 (82.7)
Re-irradiation/CyberKnife at first recurrence	20 (12.0)	5 (4.4)	15 (28.8)
Any local treatment at first recurrence	69 (41.6)	22 (19.3)	47 (90.4)
Mutually exclusive recorded first-recurrence treatment strategy			
Bevacizumab + irinotecan without recorded local therapy	97 (58.4)	97 (85.1)	0 (0.0)
Local therapy + bevacizumab + irinotecan	12 (7.2)	12 (10.5)	0 (0.0)
Bevacizumab monotherapy or unspecified bevacizumab regimen	5 (3.0)	5 (4.4)	0 (0.0)
Re-operation alone	20 (12.0)	0 (0.0)	20 (38.5)
Re-operation + temozolomide ± re-irradiation/CyberKnife	21 (12.7)	0 (0.0)	21 (40.4)
Re-irradiation/CyberKnife without surgery or systemic therapy	6 (3.6)	0 (0.0)	6 (11.5)
Temozolomide rechallenge/extended temozolomide without local therapy	4 (2.4)	0 (0.0)	4 (7.7)
Irinotecan alone	1 (0.6)	0 (0.0)	1 (1.9)

Abbreviations: IDH, isocitrate dehydrogenase; WHO, World Health Organization.

Footnote: Values are n (%). Treatment components were not mutually exclusive because patients could receive multimodal recurrence-directed therapy. Local therapy included re-operation, re-irradiation, CyberKnife, or combinations thereof. Patients were classified according to the treatment strategy recorded at first recurrence: a bevacizumab-containing strategy was defined by the presence of bevacizumab in the recorded first-recurrence regimen, whereas all other recorded first-recurrence strategies were classified as non-bevacizumab strategies. Treatment components recorded as part of the first-recurrence strategy were not assumed to have been initiated simultaneously. Subsequent receipt of bevacizumab or other treatments after the first-recurrence strategy did not change the initial treatment-strategy classification. Thus, the comparison represents the association between different recorded multimodal first-recurrence treatment strategies and should not be interpreted as isolating the pharmacologic effect of bevacizumab. Subsequent treatments after second and later progressions are reported in Table S7.

Table S7. Subsequent treatment lines and attrition after second and later progressions in the primary recurrent WHO 2021 glioblastoma, IDH-wildtype cohort

Progression line / treatment status	n (%)
Second progression status among the primary recurrent cohort, n = 166	
Documented second progression	**56 (33.7)**
Death before documented second progression	**89 (53.6)**
Alive/censored without documented second progression	**18 (10.8)**
Not evaluable for post-recurrence outcome because of insufficient or internally inconsistent follow-up	**3 (1.8)**
Treatment after valid documented second progression, n = 56	
Bevacizumab + irinotecan without recorded local therapy	**18 (32.1)**
Local therapy + bevacizumab + irinotecan	**2 (3.6)**
Re-operation ± radiotherapy/temozolomide without bevacizumab	**20 (35.7)**
Chemotherapy or targeted therapy without local therapy or bevacizumab	**9 (16.1)**
Radiotherapy/CyberKnife without surgery or systemic therapy	**2 (3.6)**
No active treatment / best supportive care	**5 (8.9)**
Third progression status among patients with valid documented second progression, n = 56	
Documented third progression	**15 (26.8)**
Death before documented third progression	**34 (60.7)**
Alive/censored without documented third progression	**7 (12.5)**
Treatment after third progression, n = 15	
Bevacizumab + irinotecan without recorded local therapy	5 (33.3)
Local therapy + bevacizumab + irinotecan	1 (6.7)
Bevacizumab monotherapy	1 (6.7)
Re-operation ± radiotherapy/temozolomide without bevacizumab	6 (40.0)
Chemotherapy without local therapy or bevacizumab	2 (13.3)
No active treatment / best supportive care	0 (0.0)
Fourth progression status among patients with documented third progression, n = 15	
Documented fourth progression	3 (20.0)
Death before documented fourth progression	10 (66.7)
Alive/censored without documented fourth progression	2 (13.3)
Treatment after fourth progression, n = 3	
Bevacizumab + irinotecan without recorded local therapy	1 (33.3)
Re-operation alone	1 (33.3)
Temozolomide without local therapy	1 (33.3)
No active treatment / best supportive care	0 (0.0)
Fifth progression status among patients with documented fourth progression, n = 3	
Documented fifth progression	0 (0.0)
Death before documented fifth progression	3 (100.0)
Alive/censored without documented fifth progression	0 (0.0)

Abbreviations: IDH, isocitrate dehydrogenase; TMZ, temozolomide; WHO, World Health Organization.

Footnote: Values are n (%). The table includes the primary recurrent WHO 2021 glioblastoma, IDH-wildtype cohort. Second-progression dates that preceded the recorded first-recurrence date were considered internally inconsistent and were not counted as documented second-progression events. Three patients lacked evaluable post-recurrence outcome information because follow-up was absent or preceded the recorded first-recurrence date and are shown separately. Percentages for progression-status rows were calculated using the number of patients at risk for the corresponding subsequent progression as the denominator. Percentages for treatment rows were calculated using the number of patients with a valid documented progression at that line as the denominator. Local therapy included re-operation, radiotherapy, CyberKnife, Gamma Knife, or combinations thereof. "Chemotherapy or targeted therapy without local therapy or bevacizumab" included carboplatin, regorafenib, nimotuzumab, temozolomide, or other non-bevacizumab systemic agents, depending on the progression line. Records coded as "no treatment" were categorized as no active treatment /

best supportive care. Because supportive-care intent was not captured as a separate structured variable, this category should be interpreted as absence of recorded active antitumor treatment rather than a prospectively defined best supportive care strategy.

Table S8. Univariable and pretreatment-adjusted Cox proportional hazards regression analyses for post-recurrence progression-free survival in the primary recurrent WHO 2021 glioblastoma, IDH-wildtype cohort

Variable	Univariable n / events	Univariable HR (95% CI)	p value	Multivariable HR (95% CI)	p value
Bevacizumab-containing first-recurrence strategy	163 / 145	**0.87 (0.61–1.23)**	**0.433**	**0.71 (0.44–1.13)**	**0.150**
Age at first recurrence, per 10-year increase	160 / 142	1.07 (0.91–1.25)	0.400	1.17 (0.97–1.42)	0.107
ECOG performance status ≥2 at diagnosis	124 / 107	1.03 (0.64–1.66)	0.913	0.96 (0.55–1.68)	0.884
Baseline NLR, per doubling	161 / 143	**1.24 (1.04–1.49)**	**0.018**	**1.43 (1.15–1.76)**	**0.001**
Time from diagnosis to first recurrence, per 6-month increase	160 / 142	0.98 (0.93–1.04)	0.553	0.98 (0.93–1.04)	0.560
Tumor size ≥5 cm	146 / 129	1.03 (0.70–1.51)	0.889	1.00 (0.64–1.58)	0.996
Multicentric disease	163 / 145	1.09 (0.70–1.70)	0.691	0.98 (0.56–1.71)	0.941
Initial non-gross total resection	153 / 136	1.47 (0.94–2.29)	0.090	1.63 (0.96–2.76)	0.071

Multivariable analysis population: n = 117; prPFS events = 100.
Abbreviations: CI, confidence interval; ECOG, Eastern Cooperative Oncology Group; HR, hazard ratio; IDH, isocitrate dehydrogenase; NLR, neutrophil-to-lymphocyte ratio; prPFS, post-recurrence progression-free survival; WHO, World Health Organization.
Footnote: prPFS was defined as the interval from first documented recurrence/progression to the earliest of documented second progression, death from any cause, or last known follow-up. Second-progression dates preceding the recorded first-recurrence date were considered internally inconsistent and were not counted as progression events. The multivariable Cox model was restricted to pretreatment characteristics and included first-recurrence treatment strategy, age at first recurrence, ECOG performance status ≥2 at diagnosis, baseline NLR modeled continuously on the log2 scale, time from diagnosis to first recurrence, tumor size ≥5 cm, multicentric disease, and initial non-gross total resection. Accordingly, the NLR HR represents the relative hazard associated with a doubling of baseline NLR. Relapse surgery and re-irradiation/CyberKnife were excluded from the primary multivariable model because they were components of the recurrence-directed treatment strategy rather than pretreatment confounders. ECOG performance status refers to the value documented at initial diagnosis because recurrence-specific ECOG/KPS data were not uniformly available. Initial non-gross total resection included biopsy and subtotal resection. The multivariable analysis was a complete-case analysis and included 117 patients with 100 prPFS events. Cox models used Breslow handling of tied event times. HRs greater than 1 indicate a higher hazard of second progression or death.

Table S9. Proportional-hazards diagnostics and time-varying-covariate sensitivity analyses for the pretreatment-adjusted Cox models

Panel A. *Proportional-hazards assumption tests*

Variable	Post-recurrence OS, p value	prPFS, p value
Bevacizumab-containing first-recurrence strategy	0.293	0.565
Age at first recurrence, per 10-year increase	0.053	0.004
ECOG performance status ≥2 at diagnosis	0.374	0.531
Baseline NLR, log2-transformed	0.136	0.005
Time from diagnosis to first recurrence, per 6-month increase	0.728	0.241
Tumor size ≥5 cm	0.001	0.175

Variable	Post-recurrence OS, p value	prPFS, p value
Multicentric disease	0.585	0.315
Initial non-gross total resection	0.766	0.645
**Global test**	**0.037**	**0.031**

Panel B. *Time-varying-covariate sensitivity analyses*

Endpoint	Covariate(s) allowed to vary with log(time)	HR for bevacizumab-containing vs non-bevacizumab strategy (95% CI)	p value	n / events
Post-recurrence OS	Tumor size ≥5 cm	1.58 (0.92–2.70)	0.096	117 / 91
prPFS	Age at first recurrence; baseline NLR, log2-transformed	0.61 (0.38–0.98)	0.041	117 / 100

Abbreviations: CI, confidence interval; ECOG, Eastern Cooperative Oncology Group; HR, hazard ratio; NLR, neutrophil-to-lymphocyte ratio; OS, overall survival; PH, proportional hazards; prPFS, post-recurrence progression-free survival.

Footnote: Values in Panel A are p values from Schoenfeld-residual-based proportional-hazards tests for the complete-case pretreatment-adjusted Cox models. The OS and prPFS models included first-recurrence treatment strategy, age at first recurrence, ECOG performance status ≥2 at diagnosis, baseline NLR modeled continuously on the log2 scale, time from diagnosis to first recurrence, tumor size ≥5 cm, multicentric disease, and initial non-gross total resection. A two-sided p value <0.05 was considered evidence against the proportional-hazards assumption. There was no evidence of non-proportional hazards for the treatment-strategy variable itself for either OS or prPFS. Because the global test indicated evidence of non-proportionality for both endpoints, sensitivity models were fitted in which covariates with significant individual PH tests were allowed to vary with log-transformed follow-up time. Accordingly, tumor size ≥5 cm was modeled as time varying for OS, whereas age at first recurrence and log2-transformed baseline NLR were modeled as time varying for prPFS. Panel B reports the treatment-strategy HR from these sensitivity models; the time-varying terms were included as nuisance terms to evaluate the robustness of the treatment-strategy association and were not interpreted as primary exposure effects. HRs greater than 1 indicate a higher hazard with a bevacizumab-containing first-recurrence strategy, whereas HRs less than 1 indicate a lower hazard.

Table S10. Covariate balance before and after pretreatment-only propensity-score overlap weighting across multiply imputed datasets

Covariate	Mean absolute SMD before weighting	Range across 50 imputations	Mean absolute SMD after overlap weighting
Age at first recurrence	0.531	0.514–0.535	<0.001
Male sex	0.050	0.050–0.050	<0.001
ECOG performance status ≥2 at diagnosis	0.083	0.008–0.255	<0.001
Baseline NLR, log2-transformed	0.015	<0.001–0.068	<0.001
Time from diagnosis to first recurrence	0.524	0.484–0.598	<0.001
Initial non-gross total resection	0.159	0.082–0.230	<0.001
Tumor size ≥5 cm	0.108	0.003–0.203	<0.001
Multicentric disease	0.045	0.045–0.045	<0.001
Tumor location, 3-level category	0.166	0.166–0.166	<0.001
Seizure at presentation	0.274	0.274–0.274	<0.001

Abbreviations: ECOG, Eastern Cooperative Oncology Group; MI, multiple imputation; NLR, neutrophil-to-lymphocyte ratio; PS, propensity score; SMD, standardized mean difference.

Footnote: Absolute standardized mean differences are summarized across 50 multiply imputed datasets in the outcome-evaluable cohort (n = 163; 113 patients receiving a bevacizumab-

containing first-recurrence strategy and 50 receiving a non-bevacizumab strategy). Propensity scores for initiation of a bevacizumab-containing first-recurrence treatment strategy were estimated separately within each imputed dataset using logistic regression. The pretreatment-only propensity-score model included age at first recurrence, sex, ECOG performance status ≥2 at diagnosis, baseline NLR modeled continuously after log2 transformation, time from diagnosis to first recurrence, initial non-gross total resection, tumor size ≥5 cm, multicentric disease, tumor location, and seizure at presentation. Relapse surgery, re-irradiation/CyberKnife, and neurological sequelae/deficit were not included in the propensity-score model. Overlap weights were calculated as 1 minus the propensity score for patients receiving a bevacizumab-containing strategy and as the propensity score for patients receiving a non-bevacizumab strategy. For the three-level tumor-location variable, the maximum absolute SMD across the three categories is reported. Pre-weighting SMDs are presented as the mean absolute SMD across the 50 imputed datasets together with the minimum-to-maximum range across imputations. Because all displayed covariates were included directly in the logistic propensity-score model, overlap weighting produced essentially exact mean balance within each imputed dataset. The largest post-weighting absolute SMD observed for any displayed covariate across all 50 imputations was less than 0.000000000000003 and is therefore reported as <0.001. An absolute SMD below 0.10 was considered indicative of adequate covariate balance.

Table S11. Propensity-score model specification, functional-form assessment, and overlap-weighting diagnostics across multiply imputed datasets

Panel A. *Propensity-score model coefficients*

Covariate	Coding / functional form	Coefficient (β)	SE	OR	95% CI	p value
Intercept	Model intercept	0.0944	1.1888	1.099	0.107–11.296	0.937
Age at first recurrence	Continuous, per 10-year increase	0.3404	0.1747	1.406	0.998–1.979	0.051
Male sex	Male vs female	−0.1322	0.3827	0.876	0.414–1.855	0.730
ECOG performance status ≥2 at diagnosis	≥2 vs 0–1	−0.0413	0.6091	0.960	0.291–3.166	0.946
Baseline NLR	Continuous log2 transformation; per doubling	0.1121	0.2019	1.119	0.753–1.662	0.579
Time from diagnosis to first recurrence	Continuous, per 6-month increase	−0.2792	0.1024	0.756	0.619–0.924	0.006
Initial non-gross total resection	Biopsy/subtotal resection vs gross total resection	−0.4913	0.5124	0.612	0.224–1.670	0.338
Tumor size ≥5 cm	≥5 cm vs <5 cm	−0.4228	0.4192	0.655	0.288–1.490	0.313
Multicentric disease	Yes vs no	−0.0311	0.5170	0.969	0.352–2.670	0.952
Tumor location: temporal	Temporal vs frontal	0.0861	0.5758	1.090	0.353–3.369	0.881
Tumor location: other	Other vs frontal	−0.3755	0.4710	0.687	0.273–1.729	0.425
Seizure at presentation	Yes vs no	−0.4274	0.4959	0.652	0.247–1.724	0.389

Panel B. *Functional-form assessment of continuous propensity-score covariates*

Continuous covariate	Final functional form	Restricted cubic spline nonlinearity test, p value
Age at first recurrence	Linear, per 10-year increase	**0.339**
Baseline NLR	Linear on the log2 scale	**0.796**
Time from diagnosis to first recurrence	Linear, per 6-month increase	**0.227**

Panel C. *Propensity-score, overlap-weight, and effective-sample-size diagnostics*

Values are mean across the 50 imputed datasets with the range across imputations in parentheses, unless otherwise specified.

Diagnostic	Bevacizumab-containing strategy, n = 113	Non-bevacizumab strategy, n = 50	Overall, n = 163
Propensity-score minimum	0.336 (0.295–0.360)	0.009 (0.004–0.016)	0.009 (0.004–0.016)
Propensity-score maximum	0.917 (0.902–0.944)	0.879 (0.858–0.933)	0.917 (0.902–0.944)
Propensity-score median*	0.771	0.648	-
Propensity-score Q1–Q3*	0.666–0.840	0.424–0.758	-
Overlap-weight minimum	0.083 (0.056–0.098)	0.009 (0.004–0.016)	0.009 (0.004–0.016)
Overlap-weight maximum	0.664 (0.640–0.705)	0.879 (0.858–0.933)	0.879 (0.858–0.933)
Effective sample size	90.4 (86.6–92.1)	43.5 (41.6–44.2)	117.4 (112.4–119.6)
Number of patients with propensity score <0.05	0 (0–0)	2.1 (2–3)	2.1 (2–3)
Number of patients with propensity score >0.95	0 (0–0)	0 (0–0)	0 (0–0)
Estimated propensity score equal to 0 or 1	0	0	0
Empirical common-support lower bound	-	-	0.336 (0.295–0.360)
Empirical common-support upper bound	-	-	0.879 (0.858–0.933)
Number of patients outside empirical common support	-	-	19.0 (9–25)

** For the propensity-score median and quartiles, values are the means of the corresponding distributional summaries across the 50 imputed datasets.*

Abbreviations: CI, confidence interval; ECOG, Eastern Cooperative Oncology Group; ESS, effective sample size; GTR, gross total resection; MI, multiple imputation; NLR, neutrophil-to-lymphocyte ratio; OR, odds ratio; PS, propensity score; Q1, first quartile; Q3, third quartile; SE, standard error.

Footnote: Propensity scores estimated the probability of initiation of a bevacizumab-containing first-recurrence treatment strategy. The propensity-score model was restricted to pretreatment characteristics and included age at first recurrence, sex, ECOG performance status ≥2 at diagnosis, baseline NLR modeled continuously on the log2 scale, time from diagnosis to first recurrence, initial non-gross total resection, tumor size ≥5 cm, multicentric disease, tumor location, and seizure at presentation. Frontal location was used as the reference category for tumor location. Relapse surgery, re-irradiation/CyberKnife, and neurological sequelae/deficit were not included in the propensity-score model.

Multiple imputation was performed in the 163 patients with evaluable post-recurrence outcomes using 50 completed datasets. The propensity-score model was re-estimated separately within each imputed dataset. Regression coefficients and their variances in Panel A were combined using Rubin's rules, and odds ratios were obtained by exponentiating the pooled coefficients. Odds ratios in Panel A describe associations with assignment to the bevacizumab-containing strategy and should not be interpreted as treatment-effect estimates.

Functional form was assessed using restricted cubic splines while retaining the remaining propensity-score covariates in the model. Four knots were placed at the 5th, 35th, 65th, and 95th percentiles of the observed non-missing distribution of each continuous covariate so that the same spline specification could be applied across all imputed datasets. The corresponding knot values were approximately 29.19, 49.45, 58.19, and 70.60 years for age at first recurrence; 1.38, 2.35, 3.82, and 12.38 for baseline NLR; and 3.18, 6.93, 13.63, and 40.08 months for time from diagnosis to first recurrence. The p values in Panel B were obtained from

pooled joint Wald tests of the nonlinear spline terms. No evidence was identified to support departure from the specified linear functional forms.
Overlap weights were calculated separately within each imputed dataset as 1 minus the propensity score for patients receiving a bevacizumab-containing strategy and as the propensity score for patients receiving a non-bevacizumab strategy. Effective sample size was calculated as the square of the sum of the weights divided by the sum of the squared weights. The overall effective sample size therefore is not the arithmetic sum of the two treatment-group-specific effective sample sizes. Propensity-score and overlap-weight diagnostics in Panel C summarize the 50 separately fitted propensity-score models. Empirical common support was defined within each imputed dataset as the interval between the larger of the two treatment-group-specific minimum propensity scores and the smaller of the two treatment-group-specific maximum propensity scores. No estimated propensity score was exactly 0 or 1. Limited empirical overlap remained in the tails of the propensity-score distributions, including a small number of patients in the non-bevacizumab strategy group with propensity scores below 0.05; overlap weighting down-weighted observations in these regions of limited overlap.

Table S12. Overlap-weighted absolute survival estimates according to first-recurrence treatment strategy

Endpoint	Time, months	Bevacizumab-containing strategy, % (95% CI)	Non-bevacizumab strategy, % (95% CI)	Absolute difference, percentage points (95% CI)
**Post-recurrence OS**	6	62.2 (51.2–73.2)	75.4 (62.3–87.6)	−13.3 (−29.3 to 4.6)
	12	40.3 (29.4–51.7)	52.5 (36.7–66.5)	−12.2 (−29.9 to 7.9)
	18	23.6 (14.7–34.0)	42.7 (28.0–56.8)	−19.2 (−36.1 to −0.7)
	24	17.6 (9.0–27.1)	32.2 (19.9–43.8)	−14.6 (−29.0 to 0.9)
**prPFS**	6	60.3 (49.2–71.4)	53.4 (38.7–67.4)	6.9 (−10.4 to 26.1)
	12	37.5 (26.9–48.5)	24.4 (12.9–36.4)	13.0 (−2.6 to 29.7)
	18	20.6 (11.8–30.9)	12.7 (4.8–22.2)	7.9 (−4.9 to 21.1)
	24	14.3 (6.2–23.4)	10.0 (2.8–18.6)	4.3 (−7.1 to 15.7)

Abbreviations: CI, confidence interval; MI, multiple imputation; OS, overall survival; prPFS, post-recurrence progression-free survival; PS, propensity score.
Footnote: Values are overlap-weighted Kaplan–Meier survival estimates. Propensity scores were based on the pretreatment-only model including age at first recurrence, sex, ECOG performance status ≥2 at diagnosis, baseline NLR modeled continuously on the log2 scale, time from diagnosis to first recurrence, initial non-gross total resection, tumor size ≥5 cm, multicentric disease, tumor location, and seizure at presentation. Multiple imputation was performed using 50 completed datasets among the 163 patients with evaluable post-recurrence outcomes. Propensity scores and overlap weights were re-estimated separately within each completed dataset. Survival probabilities shown in the table are the means of the 50 imputation-specific overlap-weighted Kaplan–Meier estimates. Absolute differences were calculated as the bevacizumab-containing strategy estimate minus the non-bevacizumab strategy estimate and are expressed in percentage points.
Ninety-five percent confidence intervals were obtained using 2,000 stratified patient-level bootstrap resamples, with resampling performed separately within the two treatment-strategy groups. The same resampled patient indices were applied to each of the 50 completed datasets, and the propensity-score model and overlap weights were re-estimated within every completed dataset in each bootstrap sample. Percentile bootstrap confidence intervals are reported. The confidence intervals were not adjusted for multiple comparisons across time points. At 24 months, the observed numbers remaining at risk were 13 versus 17 for OS and 10 versus 6 for prPFS in the bevacizumab-containing and non-bevacizumab groups, respectively; therefore,

particularly the 24-month prPFS estimates should be interpreted cautiously because of the limited late risk set.

Table S13. Overlap-weighted restricted mean survival time at 6, 12, 18, and 24 months according to first-recurrence treatment strategy

Endpoint	RMST horizon, months	Bevacizumab-containing strategy, RMST months (95% CI)	Non-bevacizumab strategy, RMST months (95% CI)	RMST difference, months (95% CI)	p value
**Post-recurrence OS**	6	4.88 (4.48–5.24)	5.08 (4.57–5.55)	−0.20 (−0.82 to 0.48)	0.542
	12	7.91 (6.95–8.85)	8.86 (7.57–10.04)	−0.95 (−2.54 to 0.67)	0.255
	18	9.77 (8.35–11.22)	11.67 (9.62–13.51)	−1.89 (−4.33 to 0.62)	0.143
	24	10.99 (9.21–12.92)	13.74 (11.07–16.18)	−2.75 (−5.92 to 0.61)	0.100
**prPFS**	6	4.82 (4.42–5.19)	4.53 (3.94–5.10)	0.29 (−0.38 to 1.00)	0.410
	12	7.60 (6.70–8.55)	6.82 (5.58–8.11)	0.78 (−0.82 to 2.38)	0.344
	18	9.35 (7.99–10.79)	8.01 (6.24–9.77)	1.34 (−1.01 to 3.65)	0.256
	24	10.41 (8.71–12.30)	8.73 (6.59–10.85)	1.69 (−1.15 to 4.55)	0.245

Abbreviations: CI, confidence interval; MI, multiple imputation; OS, overall survival; prPFS, post-recurrence progression-free survival; PS, propensity score; RMST, restricted mean survival time.

Footnote: Restricted mean survival time was estimated as the area under the corresponding overlap-weighted Kaplan–Meier survival curve from first recurrence to restriction horizons of 6, 12, 18, and 24 months. Analyses included the 163 patients with evaluable post-recurrence outcomes, comprising 113 patients receiving a bevacizumab-containing first-recurrence treatment strategy and 50 patients receiving a non-bevacizumab strategy.

Propensity scores were estimated using the pretreatment-only model including age at first recurrence, sex, ECOG performance status ≥2 at diagnosis, baseline NLR modeled continuously on the log2 scale, time from diagnosis to first recurrence, initial non-gross total resection, tumor size ≥5 cm, multicentric disease, tumor location, and seizure at presentation. Multiple imputation was performed using 50 completed datasets. Propensity scores and overlap weights were re-estimated separately within each completed dataset. RMST estimates were calculated from the imputation-specific overlap-weighted Kaplan–Meier curves and averaged across the 50 completed datasets.

RMST differences were calculated as the bevacizumab-containing strategy minus the non-bevacizumab strategy. Accordingly, a positive difference indicates more average survival or progression-free time within the specified restriction horizon with the bevacizumab-containing strategy, whereas a negative difference favors the non-bevacizumab strategy.

Ninety-five percent confidence intervals were obtained using 2,000 stratified patient-level bootstrap resamples, with resampling performed separately within treatment-strategy groups. The same resampled patient indices were applied across all 50 completed datasets, and the propensity-score model and overlap weights were re-estimated within each completed dataset for every bootstrap sample. Percentile bootstrap confidence intervals are reported. Two-sided p values were calculated using the bootstrap standard error of the RMST difference. No adjustment was made for multiple comparisons across restriction horizons because these analyses were performed as supplementary robustness analyses.

Table S14. Components of post-recurrence progression-free survival events according to first-recurrence treatment strategy

prPFS status	Overall, n = 163	Bevacizumab-containing strategy, n = 113	Non-bevacizumab strategy, n = 50
Documented second progression	56 (34.4)	23 (20.4)	33 (66.0)
Death before documented second progression	89 (54.6)	73 (64.6)	16 (32.0)
Censored without documented second progression or death	18 (11.0)	17 (15.0)	1 (2.0)
Any prPFS event	145 (89.0)	96 (85.0)	49 (98.0)

Abbreviations: IDH, isocitrate dehydrogenase; prPFS, post-recurrence progression-free survival; WHO, World Health Organization.

Footnote: Values are n (%), with percentages calculated within the corresponding outcome-evaluable treatment group. The table includes the 163 patients with evaluable post-recurrence outcomes: 113 patients receiving a bevacizumab-containing first-recurrence strategy and 50 receiving a non-bevacizumab first-recurrence strategy. Three patients from the primary recurrent cohort (n = 166) were not evaluable for post-recurrence outcomes because follow-up information was absent or preceded the recorded first-recurrence date; these comprised one patient in the bevacizumab-containing strategy group and two patients in the non-bevacizumab strategy group. prPFS was defined from first documented recurrence/progression to the earliest of documented second progression, death from any cause, or last known follow-up. Three recorded second-progression dates preceded the corresponding first-recurrence date and were considered internally inconsistent; these dates were not counted as second-progression events. All three affected patients subsequently had a valid recorded death outcome and were therefore classified as death before valid documented second progression. "Any prPFS event" comprises documented second progression or death before documented second progression. No formal between-group hypothesis testing was performed for these descriptive event components.

Table S15. Overlap-weighted competing-risk analysis of documented second progression and death before documented progression according to first-recurrence treatment strategy

Panel A. *Cumulative incidence estimates*

Competing event	Time, months	Bevacizumab-containing strategy, CIF % (95% CI)	Non-bevacizumab strategy, CIF % (95% CI)	Absolute difference, percentage points (95% CI)
**Documented second progression**	6	3.8 (0.4–8.4)	24.3 (12.6–35.9)	−20.5 (−32.8 to −8.3)
	12	11.7 (5.1–18.8)	43.7 (29.9–57.7)	−32.0 (−47.7 to −17.3)
	18	17.5 (9.4–26.3)	52.6 (37.6–67.6)	−35.0 (−52.1 to −18.2)
	24	20.2 (11.6–29.0)	55.3 (40.1–69.6)	−35.1 (−52.0 to −17.7)
**Death before documented second progression**	6	35.9 (25.6–46.6)	22.2 (10.5–35.5)	13.6 (−3.8 to 30.3)
	12	50.8 (39.6–62.2)	31.8 (18.7–46.1)	19.0 (0.4–38.0)
	18	61.9 (50.5–73.3)	34.7 (21.5–49.9)	27.1 (7.5–45.7)
	24	65.5 (54.3–77.1)	34.7 (21.5–49.9)	30.8 (11.8–48.6)

Panel B. *Cause-specific hazard analyses*

Cause-specific outcome	Events	Overlap-weighted cause-specific HR (95% CI)	p value
**Documented second progression**	56	0.29 (0.16–0.53)	<0.001
**Death before documented second progression**	89	1.65 (0.93–2.93)	0.085

Abbreviations: CI, confidence interval; CIF, cumulative incidence function; ECOG, Eastern Cooperative Oncology Group; HR, hazard ratio; MI, multiple imputation; NLR, neutrophil-to-lymphocyte ratio; PS, propensity score.

Footnote: Competing-risk analyses included the 163 patients with evaluable post-recurrence outcomes. Of these, 56 experienced a valid documented second progression, 89 died before a valid documented second progression, and 18 were censored without either event. Among patients receiving a bevacizumab-containing first-recurrence strategy, there were 23 documented second progressions, 73 deaths before documented second progression, and 17 censored observations; the corresponding numbers in the non-bevacizumab strategy group were 33, 16, and 1, respectively. Three recorded second-progression dates that preceded the first-recurrence date were considered chronologically invalid and were not counted as progression events; these patients were classified according to their subsequent valid death or censoring status.

Cumulative incidence functions were estimated using overlap-weighted Aalen–Johansen estimators, treating documented second progression and death before documented progression as mutually competing events. Propensity scores were based on the pretreatment-only model including age at first recurrence, sex, ECOG performance status ≥2 at diagnosis, baseline NLR modeled continuously on the log2 scale, time from diagnosis to first recurrence, initial non-gross total resection, tumor size ≥5 cm, multicentric disease, tumor location, and seizure at presentation. Propensity scores and overlap weights were re-estimated separately within each of the 50 completed datasets. CIF estimates shown in Panel A are the means of the 50 imputation-specific weighted estimates. Absolute differences were calculated as the bevacizumab-containing strategy minus the non-bevacizumab strategy and are expressed in percentage points.

Ninety-five percent confidence intervals for the CIFs and absolute differences were obtained using 2,000 stratified patient-level bootstrap resamples. Resampling was performed separately within the two treatment-strategy groups; the same resampled patient indices were applied to all 50 completed datasets, and the propensity-score model and overlap weights were re-estimated within every completed dataset in each bootstrap sample. Percentile bootstrap confidence intervals are reported.

Cause-specific Cox models in Panel B used the same pretreatment-only overlap weights. For the documented-progression model, deaths before progression were censored at death; for the death-before-progression model, documented progressions were censored at progression. Robust sandwich variance estimates were used within each imputed dataset and estimates were combined across the 50 datasets using Rubin's rules. HRs below 1 indicate a lower cause-specific hazard and HRs above 1 indicate a higher cause-specific hazard associated with the bevacizumab-containing strategy.

By construction, at each time point the sum of the two CIFs and the corresponding weighted prPFS survival probability equals 1; the estimates were concordant with Table S12 to numerical precision. The unweighted numbers remaining event-free and at risk at 6, 12, 18, and 24 months were respectively 67/30, 42/14, 21/8, and 10/6 in the bevacizumab-containing/non-bevacizumab groups. Therefore, the 24-month estimates should be interpreted cautiously because of the limited late risk set.

Table S16. Continuous, nonlinear, and interaction analyses of baseline neutrophil-to-lymphocyte ratio for post-recurrence outcomes

Panel A. *Prognostic association and functional form of continuous baseline NLR*

Endpoint	NLR specification	n / events	Adjusted HR (95% CI)	p value	p overall	p nonlinearity
Post-recurrence OS	Continuous NLR, per 1-unit increase	117 / 91	**1.05 (1.02–1.08)**	**<0.001**	-	-
	log2 NLR, per doubling	117 / 91	**1.49 (1.20–1.85)**	**<0.001**	-	-
	Restricted cubic spline of log2 NLR	117 / 91	-	-	**0.003**	**0.285**
prPFS	Continuous NLR, per 1-unit increase	117 / 100	**1.06 (1.03–1.08)**	**<0.001**	-	-
	log2 NLR, per doubling	117 / 100	**1.43 (1.15–1.76)**	**0.001**	-	-
	Restricted cubic spline of log2 NLR	117 / 100	-	-	**0.004**	**0.171**

Panel B. *Interaction between first-recurrence treatment strategy and continuous baseline NLR*

Endpoint	Interaction specification	n / events	Interaction ratio HR (95% CI)	p for interaction
Post-recurrence OS	Bevacizumab-containing strategy × continuous NLR	117 / 91	0.95 (0.86–1.05)	0.320
	Bevacizumab-containing strategy × log2 NLR	117 / 91	1.14 (0.72–1.80)	0.582
prPFS	Bevacizumab-containing strategy × continuous NLR	117 / 100	1.02 (0.92–1.13)	0.746
	Bevacizumab-containing strategy × log2 NLR	117 / 100	**1.64 (1.05–2.57)**	**0.030**

Panel C. *Sensitivity analysis using the historical NLR cutoff of 3.81*

Endpoint	NLR subgroup	n (Bevacizumab-containing / Non-bevacizumab)	Events, n	Adjusted HR for treatment strategy (95% CI)	p value	p for interaction
Post-recurrence OS	NLR <3.81	77 (50 / 27)	54	1.35 (0.71–2.54)	0.360	**0.447**
	NLR ≥3.81	40 (27 / 13)	37	1.96 (0.88–4.39)	0.100	-
prPFS	NLR <3.81	77 (50 / 27)	61	**0.52 (0.30–0.92)**	**0.024**	**0.065**
	NLR ≥3.81	40 (27 / 13)	39	1.29 (0.58–2.88)	0.529	-

Abbreviations: CI, confidence interval; ECOG, Eastern Cooperative Oncology Group; HR, hazard ratio; IDH, isocitrate dehydrogenase; NLR, neutrophil-to-lymphocyte ratio; OS, overall survival; prPFS, post-recurrence progression-free survival; WHO, World Health Organization.

Footnote: All analyses were performed using complete-case Cox proportional hazards models in the same pretreatment-adjusted analysis population used for the primary complete-case models. Models were adjusted for first-recurrence treatment strategy, age at first recurrence, ECOG performance status ≥2 at diagnosis, time from diagnosis to first recurrence, tumor size ≥5 cm, multicentric disease, and initial non-gross total resection, with NLR parameterized as specified in each analysis. In log2-transformed models, the HR for NLR represents the relative hazard associated with a doubling of baseline NLR. Nonlinearity was evaluated using a restricted cubic spline for log2-transformed NLR with four knots placed at the 5th, 35th, 65th, and 95th percentiles of the complete-case NLR distribution, corresponding to NLR values of approximately 1.37, 2.31, 3.79, and 12.51, respectively. Overall spline and nonlinearity p values were obtained using partial likelihood-ratio tests; the overall test compared the spline model with the corresponding model omitting NLR, whereas the nonlinearity test compared the spline model with the corresponding linear log2-NLR model. Interaction models included

the treatment-strategy-by-NLR product term together with the corresponding main effects and the same pretreatment covariates. Interaction ratio HRs quantify the multiplicative change in the treatment-strategy HR per 1-unit increase in raw NLR or per doubling of NLR in log2-transformed models. In Panel C, adjusted HRs compare the bevacizumab-containing with the non-bevacizumab first-recurrence treatment strategy within each historical NLR subgroup. The NLR cutoff of 3.81 was retained only as a sensitivity analysis because the original cutoff-selection procedure could not be independently reverified from the archived statistical documentation. Interaction analyses were exploratory, and no adjustment for multiple testing was performed. HRs >1 in the prognostic NLR analyses indicate a higher outcome hazard with increasing NLR; HRs >1 for treatment-strategy comparisons indicate a higher hazard with the bevacizumab-containing strategy.

Table S17. Sensitivity analyses for the association between the bevacizumab-containing first-recurrence treatment strategy and post-recurrence outcomes

Sensitivity analysis	Endpoint	n / events	HR for bevacizumab-containing vs non-bevacizumab strategy (95% CI)	p value
**Pretreatment-adjusted Cox model, complete-case analysis**	Post-recurrence OS	117 / 91	**1.52 (0.90–2.58)**	**0.117**
	prPFS	117 / 100	**0.71 (0.44–1.13)**	**0.150**
**Pretreatment-adjusted Cox model after MI**	Post-recurrence OS	163 / 136	**1.36 (0.88–2.09)**	**0.167**
	prPFS	163 / 145	**0.78 (0.52–1.17)**	**0.230**
**Pretreatment-only overlap-weighted Cox model, complete-case analysis**	Post-recurrence OS	117 / 91	**1.34 (0.82–2.19)**	**0.235**
	prPFS	117 / 100	**0.68 (0.43–1.08)**	**0.102**
**Pretreatment-only overlap-weighted Cox model after MI**	Post-recurrence OS	163 / 136	**1.40 (0.93–2.10)**	**0.107**
	prPFS	163 / 145	**0.81 (0.55–1.18)**	**0.267**
**Excluding tumors reclassified from previous grade 3 to WHO 2021 grade 4; pretreatment-adjusted Cox model after MI**	Post-recurrence OS	152 / 128	**1.29 (0.81–2.05)**	**0.285**
	prPFS	152 / 135	**0.83 (0.53–1.28)**	**0.394**
**Excluding tumors reclassified from previous grade 3 to WHO 2021 grade 4; pretreatment-only overlap-weighted Cox model after MI**	Post-recurrence OS	152 / 128	**1.37 (0.91–2.07)**	**0.133**
	prPFS	152 / 135	**0.87 (0.59–1.29)**	**0.503**
**Excluding early recurrence ≤6 months from diagnosis; complete-case pretreatment-adjusted Cox model**	Post-recurrence OS	82 / 64	**1.47 (0.76–2.81)**	**0.250**
	prPFS	82 / 71	**0.50 (0.28–0.89)**	**0.018**
**Excluding very early recurrence ≤3 months from diagnosis; complete-case pretreatment-adjusted Cox model**	Post-recurrence OS	112 / 87	**1.37 (0.79–2.38)**	**0.262**
	prPFS	112 / 96	**0.51 (0.31–0.84)**	**0.009**
**Bevacizumab + irinotecan-containing exposed regimens only; bevacizumab monotherapy/unspecified regimen excluded**	Post-recurrence OS	112 / 86	**1.43 (0.84–2.43)**	**0.191**

Sensitivity analysis	Endpoint	n / events	HR for bevacizumab-containing vs non-bevacizumab strategy (95% CI)	p value
	prPFS	112 / 95	**0.66 (0.41–1.07)**	**0.095**

Abbreviations: CI, confidence interval; ECOG, Eastern Cooperative Oncology Group; HR, hazard ratio; IDH, isocitrate dehydrogenase; MI, multiple imputation; NLR, neutrophil-to-lymphocyte ratio; OS, overall survival; prPFS, post-recurrence progression-free survival; PS, propensity score; WHO, World Health Organization.

Footnote: HRs compare initiation of a bevacizumab-containing first-recurrence treatment strategy with a non-bevacizumab first-recurrence strategy. The pretreatment-adjusted Cox model included treatment strategy, age at first recurrence, ECOG performance status ≥2 at diagnosis, baseline NLR modeled continuously on the log2 scale, time from diagnosis to first recurrence, tumor size ≥5 cm, multicentric disease, and initial non-gross total resection. The pretreatment-only propensity-score model included age at first recurrence, sex, ECOG performance status ≥2 at diagnosis, continuous log2-transformed baseline NLR, time from diagnosis to first recurrence, initial non-gross total resection, tumor size ≥5 cm, multicentric disease, tumor location, and seizure at presentation. Relapse surgery, re-irradiation/CyberKnife, and neurological sequelae/deficit were not included in the revised propensity-score model.

Multiple imputation by chained equations was performed using 50 imputed datasets among patients with evaluable post-recurrence outcomes. Propensity scores and overlap weights were re-estimated separately within each imputed dataset, and estimates were combined using Rubin's rules. Robust sandwich variance estimates were used for overlap-weighted Cox models. Twelve patients in the primary recurrent cohort had been reclassified from a previous grade 3 diagnosis to WHO 2021 grade 4; one lacked evaluable post-recurrence outcome information, leaving 152 outcome-evaluable patients in the corresponding exclusion analyses. A separate molecularly confirmed WHO 2021 sensitivity cohort was not constructed from the available structured variables because complete contemporary histomolecular confirmation could not be reconstructed consistently.

Early-recurrence exclusion analyses were restricted to patients with an observed and internally valid diagnosis-to-first-recurrence interval and were performed as complete-case pretreatment-adjusted Cox analyses. The regimen-restriction sensitivity analysis excluded five patients whose first-recurrence bevacizumab-containing regimen was recorded as bevacizumab monotherapy or was otherwise unspecified. The historical propensity-score model containing recurrence-directed co-treatment components was superseded by the pretreatment-only specification and was not retained as an inferential sensitivity model. Tied event times were handled using the Breslow method. HRs greater than 1 indicate a higher outcome hazard and HRs less than 1 indicate a lower outcome hazard with the bevacizumab-containing strategy.

Table S18. Recorded safety events according to first-recurrence treatment strategy in the primary recurrent WHO 2021 glioblastoma, IDH-wildtype cohort

Panel A. *Recorded safety events available by treatment strategy*

Safety event	Bevacizumab-containing strategy, n = 114	Non-bevacizumab strategy, n = 52
**Intracranial hemorrhage data available**	113/114 (99.1)	31/52 (59.6)
Intracranial hemorrhage	3/113 (2.7)	2/31 (6.5)
**Proteinuria data available**	113/114 (99.1)	30/52 (57.7)
Any proteinuria	35/113 (31.0)	11/30 (36.7)
Trace proteinuria	13/113 (11.5)	3/30 (10.0)
Proteinuria 1+	15/113 (13.3)	6/30 (20.0)
Proteinuria 2+	6/113 (5.3)	2/30 (6.7)
Proteinuria 3+	1/113 (0.9)	0/30 (0.0)
Proteinuria ≥2+	7/113 (6.2)	2/30 (6.7)
**Thromboembolic-event data available**	114/114 (100.0)	52/52 (100.0)
Any thromboembolic event	27/114 (23.7)	11/52 (21.2)
Deep vein thrombosis	12/114 (10.5)	3/52 (5.8)
Pulmonary thromboembolism	11/114 (9.6)	7/52 (13.5)
Deep vein thrombosis + pulmonary thromboembolism	3/114 (2.6)	1/52 (1.9)
Thromboembolic event, type unspecified	1/114 (0.9)	0/52 (0.0)
Treatment discontinuation due to toxicity	Not available	Not available

Panel B. *Events explicitly recorded as bevacizumab-related*

Recorded event	Bevacizumab-containing strategy
Evaluable for clinician-recorded bevacizumab-related complication	113/114 (99.1)
Any clinician-recorded bevacizumab-related complication	5/113 (4.4)
Peripheral edema	1/113 (0.9)
Liver function test elevation	1/113 (0.9)
Intracranial hemorrhage	3/113 (2.7)

Abbreviations: DVT, deep vein thrombosis; IDH, isocitrate dehydrogenase; PTE, pulmonary thromboembolism; WHO, World Health Organization.

Footnote: Values are n/N (%). Denominators reflect the number of patients with evaluable structured records for each safety variable. Ascertainment of intracranial hemorrhage and proteinuria was substantially less complete in the non-bevacizumab strategy group than in the bevacizumab-containing strategy group; therefore, these data are presented descriptively and no formal between-group hypothesis testing was performed. Thromboembolic-event status was available for all patients in both treatment groups. Events shown in Panel A represent recorded safety events and should not be interpreted as definitively treatment-attributable. Panel B includes only complications explicitly coded as bevacizumab-related in the structured dataset; an equivalent treatment-attribution variable was not available for the non-bevacizumab strategy group. Because patients received heterogeneous multimodal recurrence-directed treatment, individual adverse events cannot be causally attributed to a single treatment component using these retrospective data.
